# MiR-181a targets STING to drive PARP inhibitor resistance in *BRCA*- mutated triple-negative breast cancer and ovarian cancer

**DOI:** 10.1186/s13578-023-01151-y

**Published:** 2023-11-06

**Authors:** Matias A. Bustos, Takamichi Yokoe, Yoshiaki Shoji, Yuta Kobayashi, Shodai Mizuno, Tomohiro Murakami, Xiaoqing Zhang, Sreeja C. Sekhar, SooMin Kim, Suyeon Ryu, Matthew Knarr, Steven A. Vasilev, Analisa DiFeo, Ronny Drapkin, Dave S. B. Hoon

**Affiliations:** 1 Department of Translational Molecular Medicine, Saint John’s Cancer Institute (SJCI) at Providence Saint John’s Health Center (SJHC), 2200 Santa Monica Blvd, Santa Monica, CA 90404 USA; 2 Department of Genome Sequencing, SJCI at Providence SJHC, Santa Monica, CA 90404 USA; 3 Department of Obstetrics and Gynecology, Perelman School of Medicine, Penn Ovarian Cancer Research Center, University of Pennsylvania, Pennsylvania, PA 19104 USA; 4 Department of Gynecologic Oncology Research, SJCI at SJHC, Santa Monica, CA 90404 USA; 5 Department of Obstetrics & Gynecology, University Michigan, Ann Arbor, MI 48109 USA; 6 Department of Pathology, Rogel Cancer Center, University Michigan, Ann Arbor, MI 48109 USA

**Keywords:** PARPi, DNA damage, miR-181a-5p, TMEM173, STING, Triple-negative breast cancer, *BRCA1/2* mutations, Ovarian cancer, Extracellular microvesicles

## Abstract

**Background:**

Poly (ADP-ribose) polymerase inhibitors (PARPi) are approved for the treatment of *BRCA*-mutated breast cancer (BC), including triple-negative BC (TNBC) and ovarian cancer (OvCa). A key challenge is to identify the factors associated with PARPi resistance; although, previous studies suggest that platinum-based agents and PARPi share similar resistance mechanisms.

**Methods:**

Olaparib-resistant (OlaR) cell lines were analyzed using HTG EdgeSeq miRNA Whole Transcriptomic Analysis (WTA). Functional assays were performed in three *BRCA*-mutated TNBC cell lines. *In-silico* analysis were performed using multiple databases including The Cancer Genome Atlas, the Genotype-Tissue Expression, The Cancer Cell Line Encyclopedia, Genomics of Drug Sensitivity in Cancer, and Gene Omnibus Expression.

**Results:**

High miR-181a levels were identified in OlaR TNBC cell lines (*p* = 0.001) as well as in tumor tissues from TNBC patients (*p* = 0.001). We *hypothesized* that miR-181a downregulates the stimulator of interferon genes (STING) and the downstream proinflammatory cytokines to mediate PARPi resistance. *BRCA1* mutated TNBC cell lines with miR-181a-overexpression were more resistant to olaparib and showed downregulation in STING and the downstream genes controlled by STING. Extracellular vesicles derived from PARPi-resistant TNBC cell lines horizontally transferred miR-181a to parental cells which conferred PARPi-resistance and targeted STING. In clinical settings, STING levels were positively correlated with interferon gamma (IFNG) response scores (*p* = 0.01). In addition, low IFNG response scores were associated with worse response to neoadjuvant treatment including PARPi for high-risk HER2 negative BC patients (*p* = 0.001). OlaR TNBC cell lines showed resistance to platinum-based drugs. OvCa cell lines resistant to platinum showed resistance to olaparib. Knockout of miR-181a significantly improved olaparib sensitivity in OvCa cell lines (*p* = 0.001).

**Conclusion:**

miR-181a is a key factor controlling the STING pathway and driving PARPi and platinum-based drug resistance in TNBC and OvCa. The miR-181a-STING axis can be used as a potential marker for predicting PARPi responses in TNBC and OvCa tumors.

**Supplementary Information:**

The online version contains supplementary material available at 10.1186/s13578-023-01151-y.

## Background

Poly (ADP‑ribose) polymerase inhibitors (PARPi) promote DNA damage accumulation in tumor cells with homologous recombination (HR) deficiency, leading to the concept of synthetic lethality [1]. As a result of OlympiAD [2] and EMBRACA [3] phase III clinical trials, PARPi were approved for the treatment of patients with deleterious or suspected deleterious germline *BRCA*-mutated (*gBRCA* mt) or HER2-negative metastatic breast cancer (BC) who have been treated with chemotherapy either in the neoadjuvant, adjuvant, or metastatic settings [4]. PARPi are also indicated for ovarian, pancreatic, and prostate cancers [5]. Several clinical trials are ongoing to evaluate the efficacy of PARPi either alone or in combination with cytotoxic, targeted, or immunotherapeutic agents [6, 7]. Although PARPi has become widely used, there are still many unknowns regarding the resistance mechanisms [8]. Therefore, the identification of factors for early prediction of treatment response is needed to avoid unnecessary treatment.

Three large clinical studies, using PARPi treatment for patients with *BRCA* mt ovarian cancer (OvCa) [9, 10], concluded that the overall objective response rates (ORR) were 34% in the control group and 53.8% in the PARPi group; while in a subset of patients with platinum-resistant OvCa tumors, the ORR were 30% in the control group and 25% in the PARPi group. These results revealed that platinum-based agents and PARPi share similar mechanisms of resistance. To date, several cellular mechanisms for PARPi resistance have been reported, which include increased PARPi efflux, decreased PARP trapping, restoration of HR, and stabilization of stalled forks [11]; although, none of them are screened in the clinic as factors for intrinsic resistance to PARPi.

Recently, we reported that the downregulation of the cytosolic DNA sensor stimulator of interferon genes (STING) leads to cisplatin resistance by decreasing the expression of downstream proinflammatory cytokines in triple-negative breast cancer (TNBC) [12]. STING is a transmembrane adaptor protein that is activated by binding to cyclic guanosine monophosphate-adenosine monophosphate (cGAMP). Activated STING recruits TANK-binding kinase 1 (TBK1). Activated-TBK1 phosphorylates interferon (IFN) regulatory factor 3 (IRF3), which leads to the transcriptional activation of IFNs and other cytokines [13, 14]. This signaling pathway is triggered by cyclic GMP-AMP synthase (cGAS), a cytosolic DNA sensor that is activated by DNA damage [15].

Previously, we reported that tumor tissue *Homo sapiens* (hsa)-miR-181a-5p (or miR-181a-5p from this point on) downregulates STING and thereby allows fallopian tube secretory epithelial cells (FTSEC) to bypass interferon-mediated cell death leading to cancer cell transformation and development of high-grade serous ovarian cancer (HGSOC) [16]. High tumor miR-181a levels were also associated with decreased STING expression which correlated with a consistent decrease in interferon gamma (IFNG) response and lymphocyte infiltration in patients with HGSOC [16]. Thus, we *hypothesized* that the factors regulating STING levels modulate the downstream signaling in TNBC and OvCa and correlate with PARPi sensitivity as well as cross-resistance to platinum-based drugs.

In this study, we obtained the miR profiles of PARPi resistant cell lines using HTG EdgeSeq miRNA Whole Transcriptome Assay (HTG miR WTA) and identified that miR-181a is significantly upregulated in PARPi-resistant TNBC cell lines. Functional assays were then performed to characterize the role of miR-181a in controlling STING pathways as a mechanism of PARPi resistance. Furthermore, the *in-vitro* findings were validated using clinical specimens from TNBC and OvCa patients. This study describes a novel mechanism of PARPi resistance, as well as platinum-based drug cross-resistance; and suggests that the upregulation of miR-181a is a significant factor predicting PARPi responses in TNBC and OvCa.

## Material and methods

### Breast and ovarian cancer tissues

The study was conducted following the Declaration of Helsinki and was approved by the Ethics Committee at Saint John’s Cancer Institute (SJCI) and WIRB: MORD-RTPCR-0995. Six formalin-fixed paraffin-embedded (FFPE) OvCa tissues surgically resected were obtained from patients at the Department of Obstetrics and Gynecology, Perelman School of Medicine, U. of Pennsylvania, PA. Three tissues were treatment naïve at the time of surgery and three tissues were from a second debulking surgery after patients relapsed to adjuvant chemotherapy (Carboplatin/Paclitaxel). All the patient specimens were de-identified. Furthermore, a clinically annotated tissue microarray (TMA) for TNBC (#BR1301) was obtained from US Biomax (Derwood, MD).

### Breast and ovarian cancer cell lines

Three established human *BRCA* mt TNBC cell lines MDA-MB-436 (*BRCA1* mt), HCC1395 (*BRCA1* mt), and HCC1937 (*BRCA1* mt) were obtained from the American Type Culture Collection (ATCC, Manassas, VA) and were cultured as recommended. The establishment of HCC1937 and HCC1395 cell lines olaparib-resistant (OlaR) are described in Additional file 1: Supplementary M&M. All human cell lines have been authenticated using short tandem repeat profiling. All experiments were performed with mycoplasma-free cell lines.

### RNA isolation and RT-qPCR

Total RNA from cell lines was extracted by the Direct-zol RNA miniprep kit (#R2050, Zymo Research, Irvine, CA) according to the manufacturer’s instructions. RNA isolation from EV is described in Additional file 1: Supplementary M&M. Reverse transcription-quantitative polymerase chain reaction (RT-qPCR) was then performed for 1 ng of total RNA equivalent cDNA per RT-qPCR by a 3-step cycling protocol according to the manufacturer’s instructions. Primer sets (Integrated DNA Technologies, IA) used in RT-qPCR are shown in Additional file 2: Table S1.

### HTG EdgeSeq miRNA assay

The HTG miR WTA (HTG Molecular Diagnostics, Tucson, AR) was utilized to assess the difference in human miR transcripts between parental and OlaR cell lines using direct next-generation sequencing (NGS) as previously described [17]. HTG miR WTA was performed in triplicates for HCC1937 parental and OlaR cell lines as previously described [17].

### Plasmids

Purified lentiviral particles for STING (LPP-E1218-Lv103-050), miR-181a (LPP-HmiR0023-MR03-050-S), and their respective controls (LPP-NEG-Lv103-050, LP502-025) (GeneCopoeia, Rockville, MD) were transduced into TNBC cell lines MDA-MB-436, HCC1395, and HCC1937 as previously described [18]. Stable clones were selected with puromycin (#A11138-03, Life Technologies, Carlsbad, CA).

Knockdown experiments were performed as previously described [18]. TNBC cell lines MDA-MB-436, HCC1395, and HCC1937 were transfected with 50 nM pool siRNA targeting STING or non-targeting control (L-024333-00-0005 and D-001810-10-05, respectively, Horizon Discovery, Waterbeach, UK) using jetPRIME transfection reagent (Polypus-transfection, Illkirch, France).

### Drug treatment

Olaparib (ASD2281, Selleck Chemicals LCC, Houston, TX) was dissolved in molecular-grade water at a concentration of 10 mM. For cell viability assays, the measurements were performed after treatment with different concentrations (0, 5, 10, 15, 20, 30, 40, and 50 µM) of olaparib for 48 h, as previously described [12, 18]. For specific assays, cell lines were incubated with olaparib 10 µM for 24 and 48 h. Cisplatin (S116650MG, Selleck Chemicals) was dissolved in molecular-grade water at a concentration of 10 mM. For specific assays, cell lines were incubated with cisplatin for 12 and 24 h.

### Immunohistochemistry and miR-181a in situ hybridization

BC FFPE TMA sections were stained with STING antibody (Ab, #ab181125, Abcam, Cambridge, UK) by immunohistochemistry (IHC) as previously described [18]. Images were taken using a Mantra microscope. H-scores were calculated using QuPath v.0.3.0 (Queen's University, Belfast, Northern Ireland). Cell segmentation and quantification were conducted using QuPaths built-in “positive cell detection” as previously described [12].

*In-situ* hybridization (ISH) for miR-181a was performed on a FFPE TNBC TMA section and FFPE OvCa sections using the miRNAscope Assay (#324530, Advanced Cell Diagnostics, Newark, CA) according to the manufacturer’s instructions and as previously described [19]. MiR-181a probe (#728851-S1) was used for detecting miR-181a. Images were taken using a Mantra microscope. Cell segmentation and quantification were conducted using QuPaths built-in “cell detection” and “subcellular detection”.

### Cell viability assay

TNBC cell lines (1 × 10^3^) were seeded in a 96-well culture plate. The number of viable cells was assessed using a Cell Titer-Glo Luminescent Cell Viability assay (Promega, Madison, WI) according to the manufacturer’s instructions as previously described [12]. For assays including EV incubation, EV equivalent to 2 μg protein was added to each well, 24 h before drug treatment.

### Western blotting

Traditional western blot was performed as previously described [20], except for the antibodies that are summarized in Additional file 2: Table S1. All traditional western blot images were analyzed with ImageJ software (http://imagej.nih.gov/ij/). Western blotting was performed for CD9, CD63, and CD81 in EV derived from HCC1937 and HCC1395 parental cell lines.

Automated western blotting was performed according to the manufacturer’s protocol (Protein Simple, San Jose, CA), and quantified as previously described [18]. Protein from cell lines was extracted with lysis buffer (150 mM NaCl, 100 mM Tris–HCl pH 8, 1% NP-40, phosphatase, and protease inhibitors) and the protein concentration was adjusted to 0.5 μg/μL. Protein levels were analyzed using the Compass Software (ProteinSimple, San Jose, CA) with β-actin levels serving as the loading control. Cells were incubated with 15 μM olaparib or EVs equivalent to 100 μg protein as indicated in each experiment. Primary/secondary Ab dilutions are shown in Additional file 2: Table S1. Comparable results were obtained in traditional (Fig. 3H) and automated western blotting (Fig. 3D) for STING and β-actin proteins. Thus, automated western blotting was used for most of the western blotting to detect STING. All the uncropped western blot images were included in Additional file 1: Fig. S6–8.

### Isolation and characterization of EVs

For a detailed explanation of EV isolation please refer to Additional file 1: Supplementary M&M. Isolated EVs were analyzed for 1) size distribution and concentration using nanoparticle tracking analysis (NTA) and fluorescent Nanoparticles Tracking Analysis (FL-NTA) (EV core lab, Children’s Hospital Los Angeles, CA), and 2) protein markers using western blotting and alternating current electrokinetic platform (ACE). NTA and FL-NTA were performed as previously described [21, 22]. For a detailed explanation of characterization please refer to Additional file 1: Supplementary M&M.

### Biostatistics and bioinformatics analysis

Statistical analyses were performed using the GraphPad Prism 8 (GraphPad software, San Diego, CA) or R version 4.1.2 (https://www.R-project.org/.) in a two-tailed way. The distribution and variation within each group were assessed before statistical analysis. Two groups were compared using Student’s t-test. Multiple groups were analyzed by One-way or Two-way ANOVA followed by a post-hoc Tukey’s multiple comparisons test. The correlation between variables was determined using Pearson’s correlation test. Overall survival (OS), recurrence-free survival (RFS), and distant metastasis-free survival (DMFS) were calculated from the time of taking the first specimen until death/recurrence/distant metastasis or last contact and were analyzed using the Log-rank test. For miRNA analysis, DESeq2 normalization and statistical comparisons were performed using the HTG REVEAL software version 2.0.1. Differentially expressed miRNAs were screened using Log2 fold change (FC) > 1.5 or < − 1.5, median normalized counts > 1000. MiR expression (counts per million) was logarithmically scaled (Log10) for volcano plot data visualization. A two-sided *p* < 0.05 was considered statistically significant: *p* < 0.05, **p < 0.01, ***p < 0.001, and ns = not significant. All figures were unified using Adobe Illustrator CC (Adobe, San Jose, CA) or CorelDraw graphics suite 8X (Corel, Ottawa, Canada).

## Results

### MiR-181a levels are upregulated in olaparib resistance cell lines

**Fig. 1:**
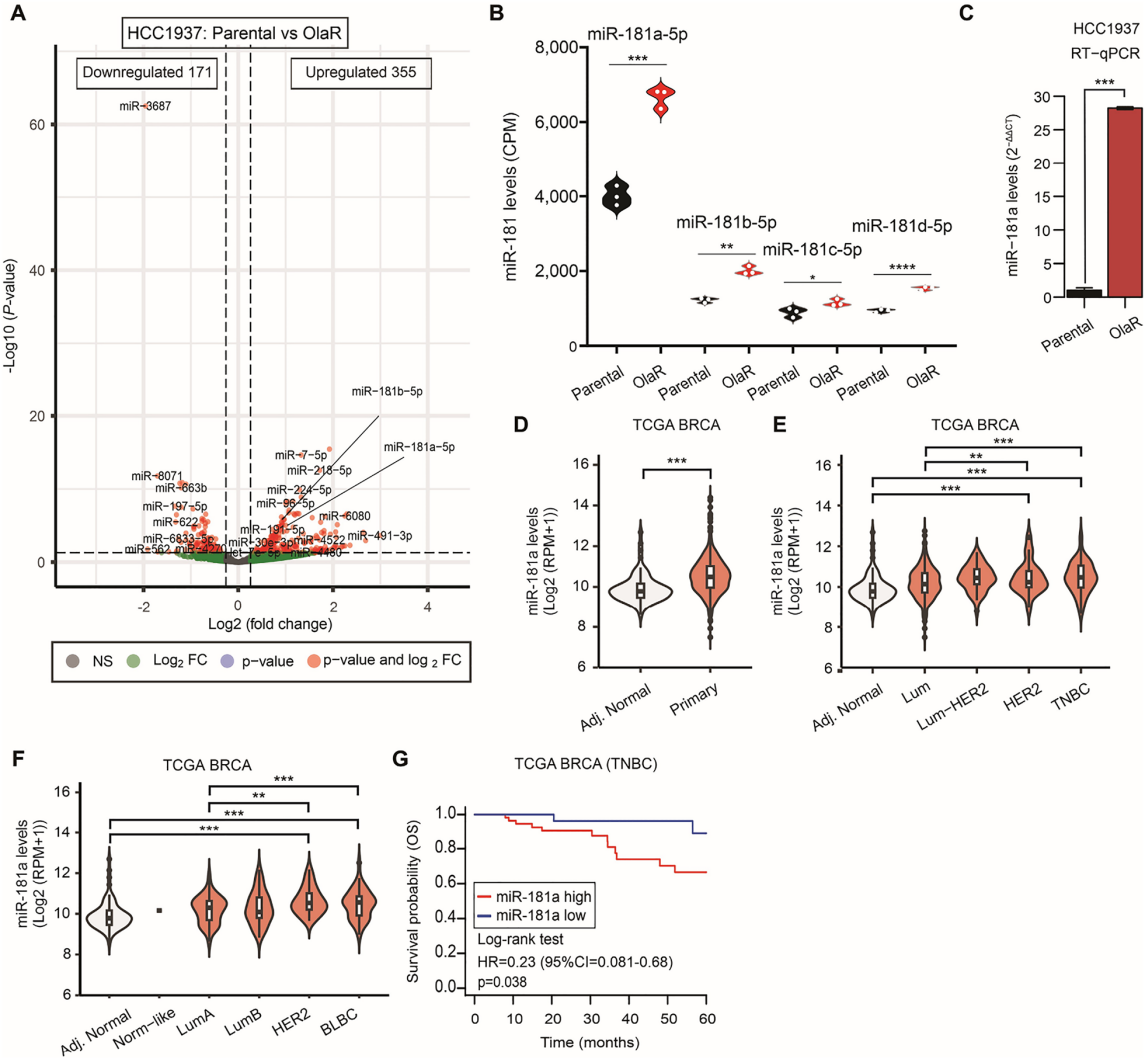
MiR-181a expression is high in Olaparib resistance TNBC cell lines. **A** Volcano plot showing the miR changes between parental and olaparib-resistant (OlaR) HCC1937 TNBC cell line using HTGq miR WTA. **B** Quantification of miR-181a, miR-181b, miR-181c, and miR-181d levels (counts per million, CPM) by HTG miR WTA in parental and OlaR HCC1937 cell line (Student’s t-test). **C** Quantification of miR-181a levels RT-qPCR (**C**) in parental and OlaR HCC1937 cell line (Student’s t-test). **D** MiR-181a levels in tumor-adjacent normal breast (Adj. Normal) and primary BC (Primary) tissues in the TCGA BRCA database (Mann–Whitney test). **E** MiR-181a levels in tissues from the tumor-adjacent normal breast (Adj. Normal), Luminal (Lum), Luminal-HER2 (Lum-HER2), HER2, and TNBC in the TCGA BRCA dataset (One-way ANOVA and Tukey’s multiple comparisons test). **F** MiR-181a levels in tissues from the tumor-adjacent normal breast (Adj. Normal), Normal-like (Norm-like), Luminal-A (LumA), Luminal-B (LumB), HER2-enriched, and basal-like breast cancer (BLBC) in the TCGA BRCA dataset (One-way ANOVA and Tukey’s multiple comparisons test). **G** Overall survival (OS) analysis for TNBC patients in the TCGA BRCA dataset (Log-rank test)

HCC1937 *BRCA1* mt olaparib-resistant (OlaR) TNBC cell line was initially established as described in Additional File 1: Supplementary M&M. Drug sensitivity assays showed that OlaR cell line was more resistant to olaparib, as well as cisplatin, than the respective parental cell line (Additional file 1: Fig. S1A, B). HCC1937 OlaR cell line showed reduced proliferation compared to the respective parental cell line (Additional file 1: Fig. S1C). HTG miR WTA was then performed to unveil genome-wide miR transcriptome changes between parental and OlaR cell lines. A total of 165 miRs were differentially expressed (DE) in the HCC1937 OlaR compared to parental cell lines (Log2 Fold Change (FC) > 1.5; < − 1.5, False Rate Discovery (FDR) < 0.01 (Fig. 1A). Only miRs upregulated and highly detected in the assay (median normalized counts > 1000) were considered significant and as potential drivers of PARPi resistance (Table 1). All the miR-181 family members, miR-181a-5p and miR-181b-5p levels -which are located in the chromosome 1q32 region [23]- and miR-181c-5p and miR-181d-5p -which are located in chromosome 19p13.2 region- were significantly increased in OlaR compared to respective parental cell lines. Although, all the members of miR-181 family shared the same seed sequence and have the potential to target STING, we decided to focus on miR-181a-5p as the detection levels were higher than miR-181b-5p, miR-181c-5p, and miR-181d-5p in the OlaR cell line (Fig. 1B); which is consistent with our previous observations in HGSOC tumors [16]. To validate the HTG miR WTA results, the miR-181a-5p levels were assessed by RT-qPCR. MiR-181a-5p showed a significantly higher expression in HCC1937 OlaR compared to the respective parental cell lines (Fig. 1C). In summary, miR-181a is upregulated in TNBC OlaR compared to respective parental cell lines. For simplification purposes miR-181a-5p from here on will be referred to as miR-181a.

**Table 1:** Differentially expressed miRNAs using HTG EdgeSeq miRNA Whole Transcriptome Assay for HCC1937 Parental vs OlaR^1^ cell line

Probe	Parental (mean normalized)	OlaR (mean normalized)	FC^2^	Adj. p-value
miR-16-5p	5760	12156	2.09	8.39E-06
let-7f-5p	4044	7922	1.94	4.04E-05
miR-25-3p	1070	2091	1.93	1.00E-03
miR-181a-5p	2707	5193	1.90	1.83E-04
miR-21-5p	20160	38679	1.90	9.10E-05
miR-151a-3p	1006	1836	1.81	2.00E-03
miR-107	3335	5831	1.73	1.30E-03
miR-29a-3p	6243	10896	1.73	4.10E-03
miR-106b-5p	1631	2784	1.69	1.53E-02
miR-93-5p	2204	3728	1.68	2.50E-03
miR-103a-3p	3979	6674	1.67	2.60E-03
miR-191-5p	1818	3013	1.65	5.42E-04
miR-22-3p	4394	7262	1.64	1.28E-02
miR-29b-3p	8474	13981	1.64	3.70E-03
let-7d-5p	4211	6891	1.62	6.80E-03
miR-30b-5p	2580	4173	1.61	3.50E-03
miR-200c-3p	6711	10725	1.59	1.23E-02
miR-31-5p	9861	15412	1.55	2.90E-02
miR-99b-5p	1245	1929	1.54	9.70E-03
let-7i-5p	1781	2711	1.51	2.18E-02
miR-2861	5604	3681	− 1.51	9.70E-03
miR-5585-3p	2666	1710	− 1.55	1.93E-02
miR-6780b-5p	1745	1112	− 1.56	7.39E-05
miR-6088	3439	2132	− 1.61	1.02E-05
miR-6126	9182	5629	− 1.62	1.07E-04
miR-6803-5p	2051	1241	− 1.64	9.73E-04
miR-3687	6336	1622	− 3.88	2.62E-60

^1^*OlaR* Olaparib- resistant, ^2^*FC* fold change

### Increased miR-181a levels were associated with worse outcomes in TNBC tumors

Based on the HTG miR WTA results, we analyzed the associations between the miR-181a levels and the histological subtype, the molecular classification for BC subtypes, and disease outcomes in TNBC patients. *In-silico* analysis using the TCGA BRCA dataset showed that miR-181a levels were significantly higher in primary BC compared to adjacent normal breast tissues (Fig. 1D). In the TCGA BRCA dataset, miR-181a levels were compared across the subtypes and adjacent normal tissues. TNBC tissues showed significantly higher miR-181a levels compared to adjacent normal and luminal BC tissues (Fig. 1E). In assessing PAM50 classification, a 50-gene signature that classifies breast cancer into five molecular intrinsic subtypes, miR-181a levels were significantly higher in basal-like BC (BLBC) compared to adjacent normal and luminal-A BC tissues (Fig. 1F). TNBC patients with high miR-181a levels showed a significantly shorter overall survival (OS) (*p* = 0.038) (Fig. 1G). In summary, miR-181a is significantly upregulated in OlaR cell lines and TNBC tumors and is associated with worse outcomes in patients with TNBC.

### MiR-181a overexpression leads to PARP inhibitor resistance by targeting STING

**Fig. 2:**
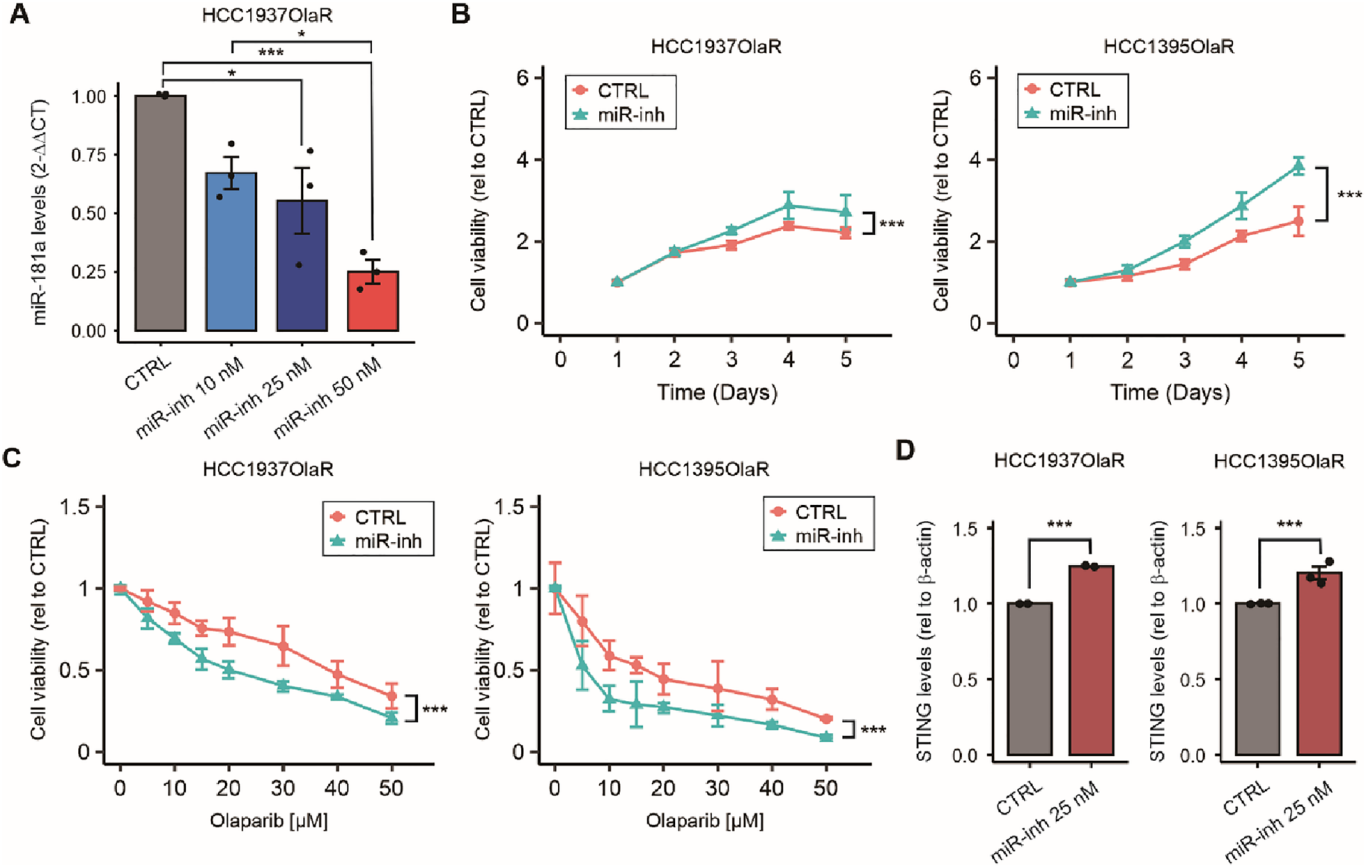
Targeting miR-181a increased olaparib sensitivity and increased STING protein levels. **A** Quantification of miR-181a levels by RT-qPCR comparing HCC1937 OlaR cell line treated with miR-181a-hairpin inhibitor (miR-inh, 10 nM, 25 nM, and 50 nM) or with miR hairpin inhibitor negative control (CTRL) in HCC1937-OlaR cell line (One-way ANOVA). **B** Cell viability assays comparing miR-181a-inhibitor and miR hairpin inhibitor negative control (CTRL) in HCC1937-OlaR and HCC1395-OlaR cell lines (two-way ANOVA and Sidak’s multiple comparisons test). **C.** Drug sensitivity assays comparing miR-181a-inhibitor and miR hairpin inhibitor negative control (CTRL) in HCC1937-OlaR and HCC1395-OlaR cell lines, treated with different concentrations of olaparib (two-way ANOVA and Sidak’s multiple comparisons test). **D** Quantification of Western blotting analysis for STING and β-actin (loading control) comparing HCC1937 OlaR and HCC1395 OlaR cell lines treated with miR-181a-inhibitor or miR hairpin inhibitor negative control (CTRL). *p < 0.05, **p < 0.01, ***p < 0.001. Cell viability assays were performed in triplicates

To further characterize the role of miR-181a, two OlaR TNBC g*BRCA* mt cell lines were treated with the miR-181a inhibitor. The results demonstrated that decreasing the endogenous levels of miR-181a increased OlaR TNBC cell line proliferation, but also increased the sensitivity to PARPi (Fig. 2A–C). Previous studies by our group found increased miR-181a levels in FTSEC and demonstrated that miR-181a targets STING [16]. Moreover, STING suppression leads to cisplatin-resistance in TNBC [12]. Since platinum agents and PARPi are suggested to share similar mechanisms of resistance [9, 10], we aimed to (i) identify the interaction of miR-181a and STING, (ii) the role of STING in promoting PARPi resistance, and (iii) the potential cross-resistance with platinum-drugs in TNBC cell lines.

**Fig. 3:**
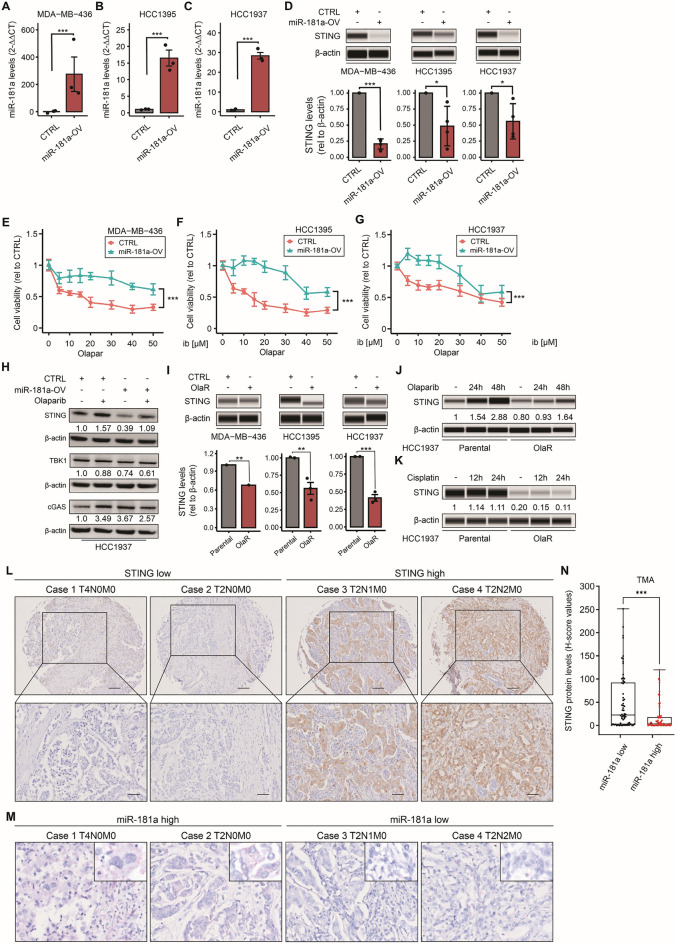
MiR-181a overexpression leads to Olaparib resistance and downregulates STING. **A**–**C** Quantification by RT-qPCR of miR-181a levels in miR-181a-OV and empty vector (CTRL) MDA-MB-436 (**A**), HCC1395 (**B**), and HCC1937 (**C**) cell lines (Student’s t-test). **D** Western blotting analysis for STING and β-actin (loading control) comparing miR-181a-OV and empty vector (CTRL) in MDA-MB-436, HCC1395, and HCC1937 cell lines. **E**–**G** Drug sensitivity assays comparing miR-181a-OV and empty vector (CTRL) in MDA-MB-436 (**E**), HCC1395 (**F**), and HCC1937 (**G**) cell lines, treated with different concentrations of olaparib (Two-way ANOVA and Sidak’s multiple comparisons test). **H** Western blotting analysis for STING, TBK1, cGAS, and β-actin (loading control), comparing miR-181a-OV and empty vector (CTRL) HCC1937 cell lines with or without olaparib treatment. **I** Western blotting analysis for STING and β-actin (loading control) comparing parental and OlaR in MDA-MB-436, HCC1395, and HCC1937 cell lines. **J** Western blotting analysis for STING and β-actin (loading control) comparing parental and OlaR HCC1937 cell line in resting and olaparib-treated (24 and 48 h) conditions. **K** Western blotting analysis for STING and β-actin (loading control) comparing parental and OlaR HCC1937 cell line in resting and cisplatin-treated (12 and 24 h) conditions. **L**, **M** Representative images of STING IHC (**L**) and miR-181a ISH (**M**) for TNBC tumor tissues in the TMA. Images of cases 1 and 2 (STING low, miR-181a high) and cases 3 and 4 (STING high, miR-181a low). **N** Comparison of STING protein levels in low versus high miR-181a TNBC tumor tissues in the TMA (Student’s t-test). *p < 0.05, **p < 0.01, ***p < 0.001. Cell viability assays were performed in triplicates

To determine if miR-181a plays a role in decreasing STING mRNA levels, we treated OlaR TNBC *gBRCA1* mt cell lines with miR-181a hairpin anti-miR. In western blot analysis, STING protein levels significantly increased upon miR-181a inhibition (Fig. 2D). Based on these results, we established three TNBC g*BRCA1* mt cell lines (HCC1937, HCC1395, and MDA-MB-436) with miR-181a overexpression (miR-181a-OV, Fig. 3A–C and Additional file 1: Fig. S1D). As expected, STING mRNA and protein levels were significantly lower in miR-181a-OV compared to parental cell lines (Additional file 1: Fig. S4K, P and Fig. 3D, respectively). MiR-181a-OV did not affect cellular proliferation (Additional file 1: Fig. S1E–G) but induced PARPi resistance in the three TNBC g*BRCA1* mt cell lines (Fig. 3E–G). Then, miR-181a-OV cell lines were treated with olaparib and compared to control cell lines. Olaparib treatment increased STING protein levels in both HCC1937 control and miR-181a-OV cell lines compared to untreated conditions. Overall STING protein levels were reduced in miR-181a-OV compared to control cell lines under untreated and treated conditions (Fig. 3H). In summary, miR-181a-OV reduced STING mRNA and protein levels in resting conditions and olaparib-treated conditions, and induced PARPi resistance in TNBC g*BRCA1* mt cell lines.

Next, we analyzed STING protein levels in OlaR cell lines. Of notice, STING protein levels were significantly lower in OlaR compared to parental cell lines (Fig. 3I). We also performed western blot analysis to compare STING protein levels between HCC1937 OlaR and parental TNBC cell lines in non-treated and olaparib-treated conditions. Olaparib treatment increased STING protein levels over treatment time in both cell lines (Fig. 3J); however, STING protein levels were significantly lower in the HCC1937 OlaR cell line in both non-treated and olaparib-treated conditions (Fig. 3J). In summary, PARPi resistant TNBC cell lines show reduced STING protein levels in non-treated and olaparib treated conditions, thus, validating our previous observations in miR-181a-OV cell lines.

In addition, serial TNBC tissue samples from a TMA were analyzed for STING by IHC and for miR-181a-5p by in-situ hybridization using miRNAscope. The results clearly showed a negative association between miR-181a and STING protein levels in TNBC tissue samples (Fig. 3L–N).

### MiR-181a upregulation induces platinum-drug cross-resistance

Two major clinical trials have shown that platinum agents and PARPi share similar mechanisms of resistance in *gBRCA* mt OvCa [9, 10]. In a previous study Knarr et al. [16], demonstrated that OvCa cell lines with enhanced miR-181a levels were significantly more resistant to platinum-based agents. Here, we used cell viability assays to show that TNBC OlaR cell lines were more resistant to cisplatin treatment compared to parental cell lines (Additional file 1: Fig. S1B); thus, confirming the cross-resistance to cisplatin. Similar results were obtained in TNBC cell lines with miR-181a-OV (Additional file 1: Fig. S1H). Then, western blotting analysis was performed to compare STING protein levels between OlaR and parental TNBC cell lines treated with cisplatin. The rationale is that miR-181a upregulation in OlaR may have implications in platinum-based agents cross-resistance by targeting STING. The results showed that STING protein levels were significantly lower in the OlaR cell line under both resting and cisplatin-treated conditions (Fig. 3K). During cisplatin treatment, STING protein levels did not change in parental or OlaR TNBC cell lines (Fig. 3K). To summarize, OlaR and miR-181a-OV TNBC cell lines showed cisplatin resistance.

### STING downregulation leads to PARPi resistance in TNBC

**Fig. 4:**
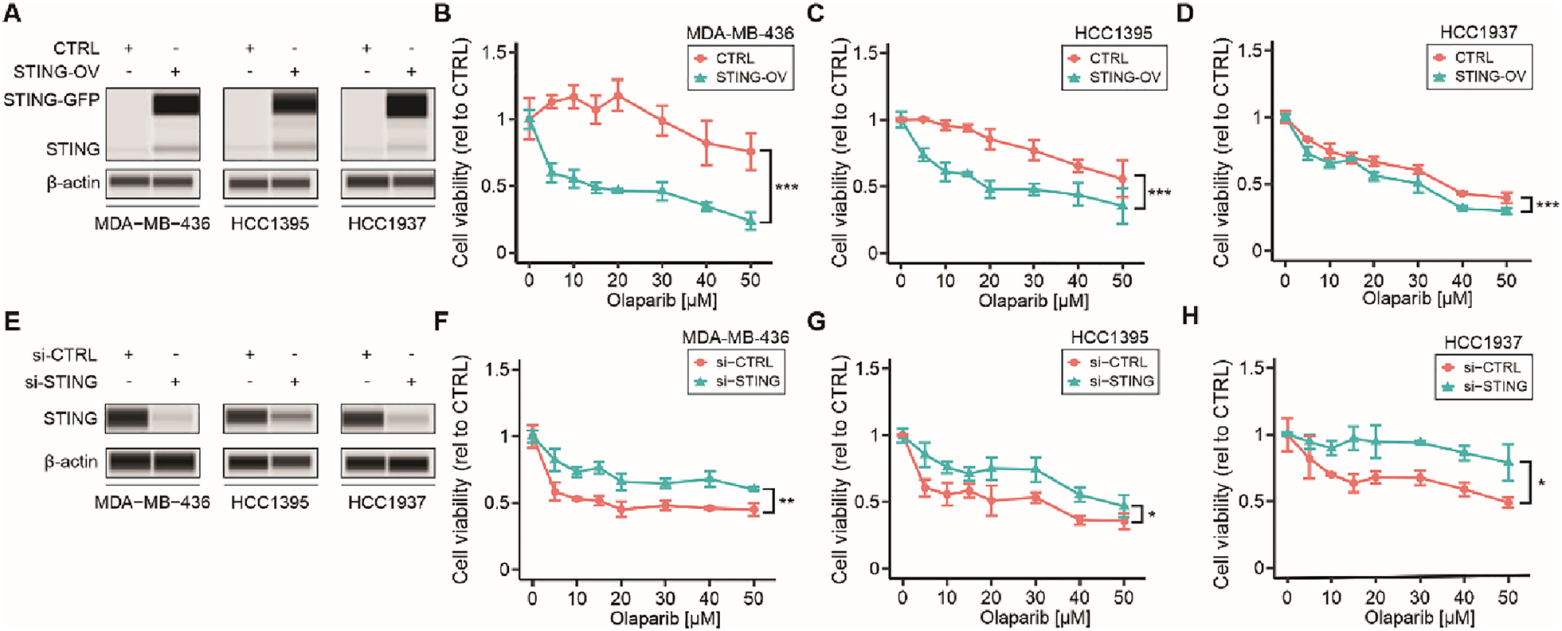
STING downregulation leads to PARPi resistance in TNBC. **A** Western blotting analysis for STING-GFP, STING, and β-actin (loading control) comparing STING-OV and empty vector (CTRL) in MDA-MB-436, HCC1395, and HCC1937 cell lines. **B**–**D** Drug sensitivity assays comparing STING-OV and empty vector (CTRL) in MDA-MB-436 (**C**), HCC1395 (**D**), and HCC1937 (**E**), treated with different concentrations of olaparib (Two-way ANOVA and Sidak’s multiple comparisons test). **E** Western blotting analysis for STING and β-actin (loading control) comparing si-STING and the respective si-CTRL in three *BRCA* mt TNBC cell lines MDA-MB-436, HCC1395, and HCC1937. **F**–**H** Drug sensitivity assays comparing si-STING and si-CTRL in MDA-MB-436 (**F**), HCC1395 (**G**), and HCC1937 (**H**) cell lines, treated with different concentrations of olaparib (Two-way ANOVA and Sidak’s multiple comparisons test). *p < 0.05, **p < 0.01, ***p < 0.001. Cell viability assays were performed in triplicates

To confirm the relation between STING levels and PARPi sensitivity, STING-overexpression (OV) was performed in the three TNBC cell lines using lentiviral transduction (Additional file 1: Fig. S2A). STING-OV was confirmed by western blotting (Fig. 4A). STING-OV did not affect cellular proliferation (Additional file 1: Fig. S2B–D**)** but increased PARPi sensitivity in *BRCA1* mt TNBC cell lines significantly (Fig. 4B–D). To further confirm the role of STING during PARPi treatment, STING knockdown was performed in three *BRCA1* mt TNBC cell lines. STING knockdown was confirmed by western blotting (Fig. 4E). STING downregulation showed no effect on cellular proliferation in *BRCA1* mt TNBC cell lines (Additional file 1: Fig. S2E–G), however, it triggered PARPi resistance in all three *BRCA* mt TNBC cell lines (Fig. 4F–H). In summary, high STING levels increased the sensitivity to PARPi while low STING levels are associated with PARPi resistance in g*BRCA1* mt TNBC.

To support these results, *in-silico* analyses were conducted on the *STING* mRNA levels in TCGA BRCA and GTEx datasets. *STING* mRNA levels were significantly lower in primary BC with invasive ductal carcinoma (IDC) compared to normal breast and adjacent normal breast tissues (Additional file 1: Fig. S3A). The patients included in the TCGA BRCA dataset were stratified into the different BC subtypes based on the molecular subtypes and PAM50 classification. *STING* mRNA levels were compared across the subtypes and adjacent normal tissues. TNBC and BLBC tissues showed significantly lower *STING* mRNA levels compared to adjacent normal and luminal BC tissues (Additional file 1: Fig. S3B–C).

Survival analyses were performed using TCGA, GEO, and EGA databases. TNBC patients with low *STING* mRNA levels showed significantly reduced RFS, DMFS, and OS (Additional file 1: Fig. S3D–F). Comparable results were observed in the subset of patients with BLBC subtype **(**Additional file 1: Fig. S3G–I). In summary, *STING* mRNA levels were significantly downregulated in TNBC tumors and low *STING* mRNA levels were associated with significantly worse survival outcomes in patients with TNBC or BLBC subtype.

### MiR-181a decreases the levels of pro-inflammatory cytokines and IFNG responses during PARPi treatment

**Fig. 5:**
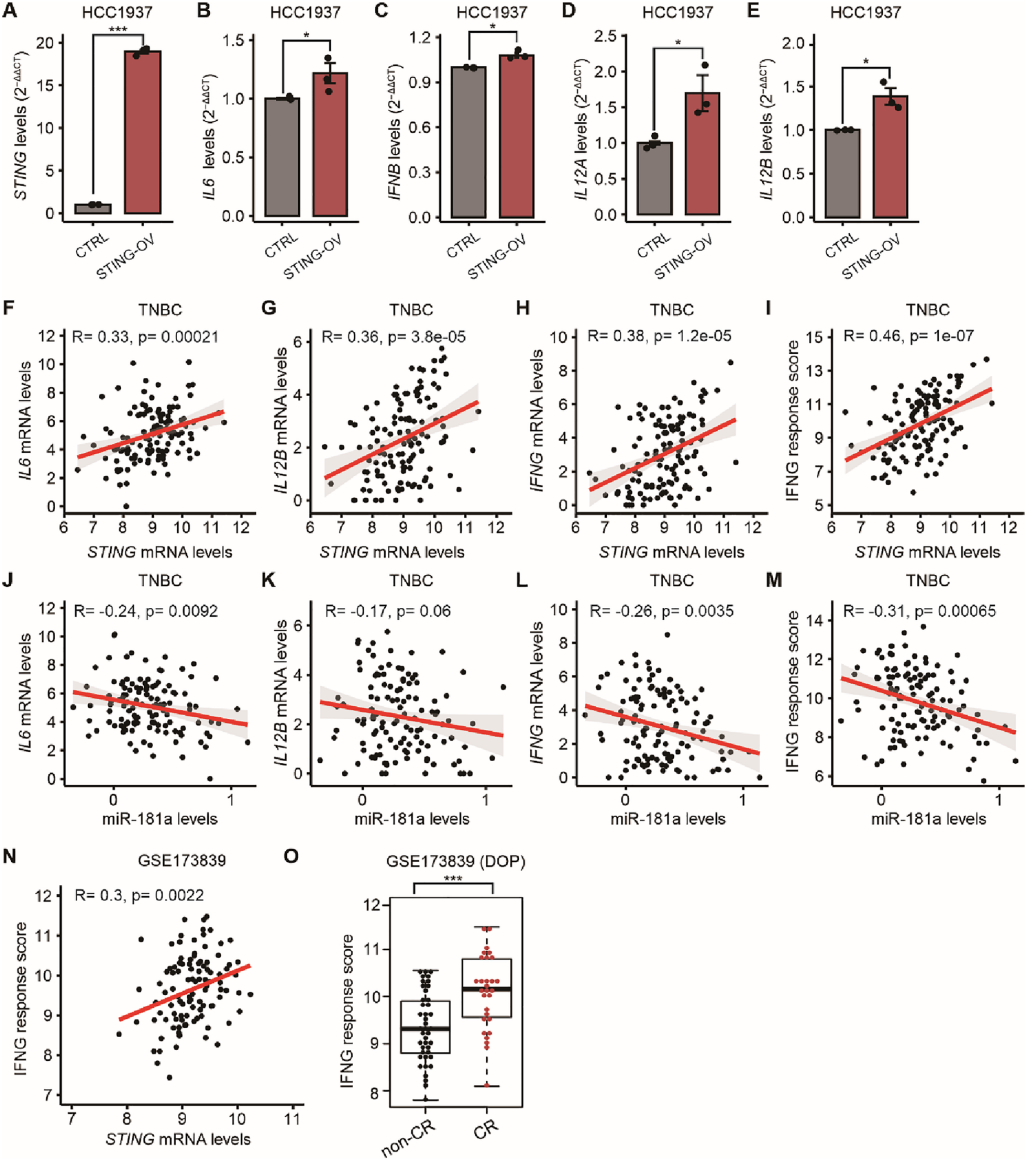
Suppression of pro-inflammatory cytokines and IFNG responses mediated by miR-181a are associated with worse PARPi responses. **A**–**E** Quantification of *STING* (**A**), *IL6* (**B**), *IFNB* (**C**), *IL12A* (**D**), and *IL12B* (**E**) mRNA levels in empty vector (CTRL) and STING-OV HCC1937 cell line by RT-qPCR (Student’s t-test). **B**–**I** Correlation between miR-181a levels and *IL6* (**F**), *IL12B* (**G**), and *IFNG* (**H**) mRNA levels; and IFNG response score (**I**) in TNBC in the TCGA BRCA database (Pearson’s correlation coefficient). **J**–**M** Correlation between *STING* mRNA levels and *IL6* (**J**), *IL12B* (**K**), and *IFNG* (**L**) mRNA levels; and IFNG response score (**M**) in TNBC in the TCGA BRCA database (Pearson’s correlation coefficient). **N** Correlation between IFNG response score and STING mRNA levels in the pretreatment biopsy tissues from patients enrolled in the I-SPY2 trial (Pearson’s correlation coefficient). **O** Comparison of IFNG response score in the pretreatment biopsy tissues of patients who underwent durvalumab/olaparib/paclitaxel (DOP) and had complete response (CR) or non-CR, in the I-SPY2 trial (Student’s t-test). *p < 0.05, **p < 0.01, ***p < 0.001

In a previous study, we demonstrated that high miR-181a/low STING levels bypass IFN-mediated cell death in FTSEC [16]. Therefore, we hypothesized that miR-181a-mediated STING suppression leads to the downregulation of the downstream pro-inflammatory cytokine genes and interferon gamma IFNG responses, thereby inducing PARPi resistance in TNBC. Three TNBC cell lines with STING-OV showed significantly higher mRNA levels of interleukin-6 (*IL6)*, *IFNB*, interleukin-12A (*IL12A)*, and interleukin-12 (*IL12B)* compared to respective control cell lines in RT-qPCR analysis (Fig. 5A–E**, **Additional file 1: Fig. S4A–J). Supporting these findings, *in-silico* analysis using the TCGA BRCA dataset demonstrated a significant positive correlation between *STING* mRNA levels and *IL6* and *IL12B*, in a subset of tissues from patients with TNBC subtype (Fig. 5F–G). In addition, TNBC patients had a significant positive correlation between *STING* and *IFNG* mRNA levels, as well as *STING* and the IFNG response scores (Fig. 5H–I).

The correlations between miR-181a and the STING downstream proinflammatory cytokine genes were analyzed by RT-qPCR in TNBC cell lines and further confirmed using the TCGA BRCA dataset. In vitro, two TNBC cell lines with miR-181a-OV showed significantly lower expression of *IL6*, *IFNB*, *IL12A*, and *IL12B* compared to the control cell lines (Additional file 1: Fig. S4L-O, Q-T). A significant negative correlation was observed between miR-181a levels and *IL6* and *IL12B* in tumor tissues from patients diagnosed with TNBC (Fig. 5J–K). Also, TNBC patients had a significant negative correlation between miR-181a levels and *IFNG* mRNA levels, as well as the IFNG response scores in tumor tissues from TNBC patients (Fig. 5L–M). Furthermore, the correlation between *STING* mRNA levels and IFNG response scores, as well as the impact of IFNG scores in predicting PARPi response, were analyzed in pretreatment FFPE tissue biopsies from patients who were enrolled in the I-SPY2 trial [24]. Using this dataset, the *STING* mRNA levels were significantly and positively correlated with IFNG response scores (Fig. 5N). Also, the IFNG response scores were significantly higher in a subset of 71 patients who received durvalumab, olaparib, and paclitaxel in neoadjuvant treatment and achieved complete response (CR) compared to patients with non-CR (Fig. 5O). In summary, miR-181a-mediated STING suppression leads to significantly decreased *IL6* and *IL12B* cytokines and reduced IFNG responses. The downregulation of the *IL6* and *IL12B* cytokines and the IFNG responses in TNBC tumor samples were associated with significantly poor responses to neoadjuvant therapies that included olaparib.

### EVs confer PARPi resistance by horizontal transfer

**Fig. 6:**
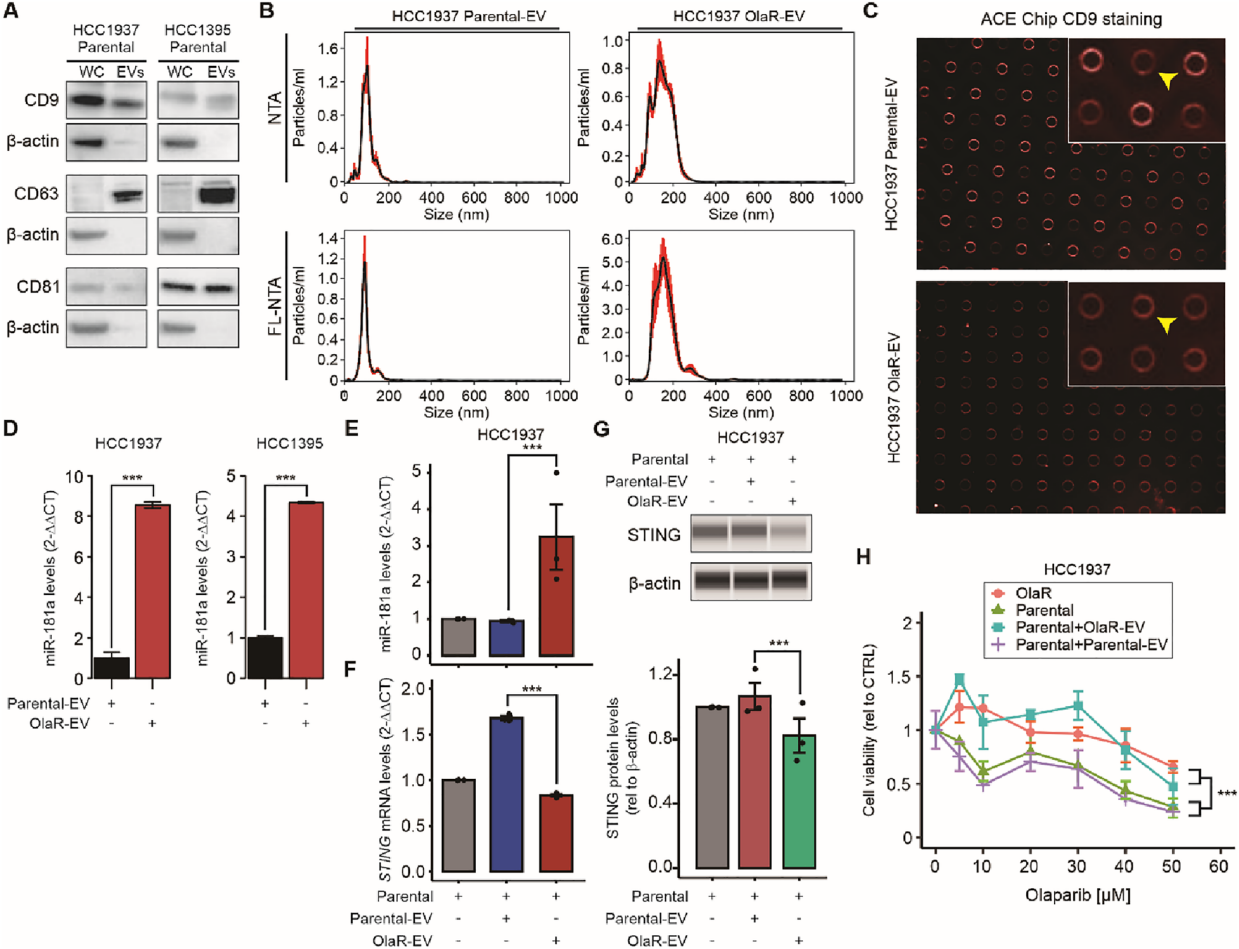
EVs transfer miR-181a and confer PARPi resistance. **A** Western blotting analysis of the tetraspanins CD9, CD63, and CD81 in whole cell extracts (WC) and extracellular vesicles (EVs) collected from HCC1937 and HCC1395 parental cell lines. **B**–**C** Characterization of HCC1937 parental cell line derived EV (parental-EV) and HCC1937 OlaR cell line derived EV (OlaR-EV) by NTA and FL-NTA (**B**) or ACE (**C**). The yellow arrowhead indicates the EVs that were bound to the Chip and stained by labeled anti-CD9 antibody. **D** Quantification of miR-181a levels by RT-qPCR comparing isolated EVs from parental (parental-EV) and OlaR (OlaR-EVs) cell lines (Student’s t-test). **E**–**F** Quantification of miR-181a (**E**) and *STING* mRNA (**F**) levels by RT-qPCR comparing HCC1937 parental cell line untreated or incubated with parental-EVs or OlaR-EVs (One-way ANOVA). **G.** Western blotting analysis for STING and β-actin (loading control) comparing HCC1937 parental cell line untreated or incubated with parental-EVs or OlaR-EVs. **H** Drug sensitivity assays comparing HCC1937 parental and OlaR cell lines incubated with parental-EVs or OlaR-EVs and treated with different concentrations of olaparib (One-way ANOVA and Sidak’s multiple comparisons test). *p < 0.05, **p < 0.01, ***p < 0.001. Cell viability assays were performed in triplicates

We investigated in vitro whether miR181a can be horizontally transferred from OlaR TNBC cell lines as a mechanism to transmit PARPi resistance to non-resistant cell lines. We focused on lipid bound extracellular vesicles (EVs) as the vehicle for miR-181a delivery to non-resistant cell lines. EVs derived from HCC1937 TNBC parental (parental-EV) and OlaR (OlaR-EV) cell lines were isolated using differential ultracentrifugation (DUC) from culture supernatants. EVs were characterized using Western blotting, NTA, FL-NTA, and ACE. By western blot, the three EVs standard markers (CD9, CD63, and CD81) were detected, indicating the purity and characteristics of the EV fractions isolated (Fig. 6A). FL-NTA analysis revealed that the size of EVs isolated by DUC was between 130 and 200 nm (Fig. 6B). Fluorescence imaging for CD9 positive EVs isolation was performed utilizing ACE Chip. The results indicated that a high proportion of CD9 positive EV were isolated by DUC (Fig. 6C).

Total RNA was extracted from EVs and the miR-181a levels were assessed using RT-qPCR. MiR-181a levels were significantly higher in OlaR-EVs compared to parental-EVs isolated from two OlaR TNBC cell lines (Fig. 6D); thus, validating our observations that OlaR cell lines showed significantly higher levels of miR-181a compared to respective parental cell lines, but also demonstrating that the miR-181a can be released in EVs. Since miR-181a is released in EVs, we hypothesized that OlaR cell lines may release these EVs to transfer resistance to adjacent non-resistant cancer cells as a mechanism of propagation of PARPi resistance. To verify this hypothesis, HCC1937 parental cell lines were incubated with isolated parental-EV, or OlaR-EV, or left untreated. Incubation with OlaR-EVs significantly increased miR-181a levels in parental cell lines (Fig. 6E). In addition, RT-qPCR as well as western blot analysis confirmed that the incubation with OlaR-EV significantly reduced STING mRNA and protein levels (Fig. 6F, G). Furthermore, TNBC parental cell lines incubated with OlaR-EVs were more resistant to olaparib compared to parental cell lines incubated with control-EV (Fig. 6H). In summary, EVs derived from HCC1937 OlaR TNBC cell lines are enriched with miR-181a. In vitro incubation of non-resistant TNBC cell lines with OlaR-EVs promotes horizontal transfer of miR-181a, decreased STING levels, and induced olaparib resistance to non-resistance TNBC cells.

### MiR-181a is upregulated in OvCa tumor tissues that failed adjuvant treatment

**Fig. 7:**
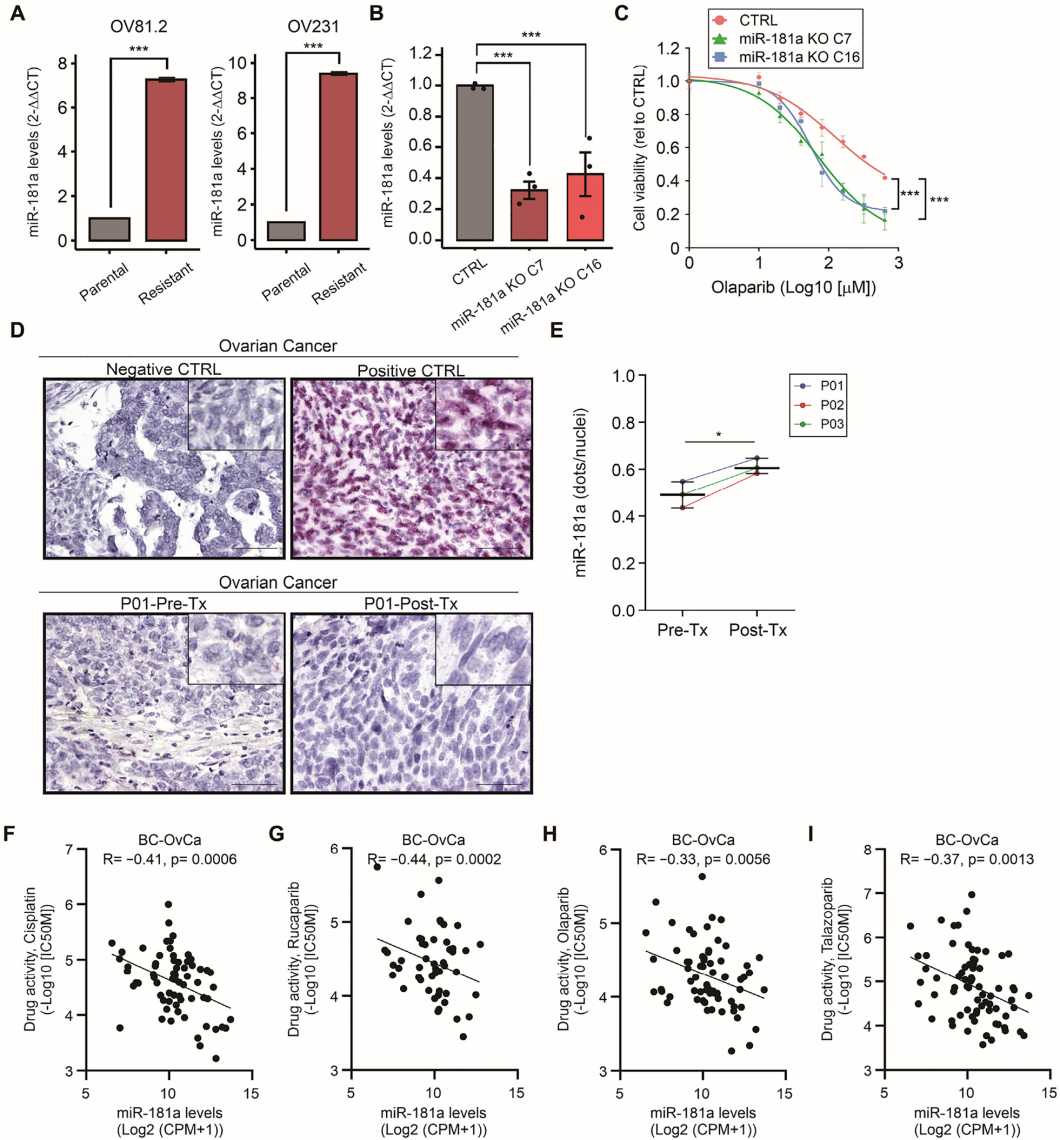
High miR-181a levels in OvCa FFPE tissues are associated with olaparib resistance. **A** Quantification of miR-181a levels by RT-qPCR comparing parental and cisplatin- and olaparib-resistant (Resistant) OV81.2 and OV231 cell lines (Student’s t-test). **B** Quantification of miR-181a levels by RT-qPCR comparing control Clone 1 (CTRL), miR-181a KO Clone 7 (C7), and miR-181a KO Clone 16 (C16) cell lines (One-way ANOVA). **C** Drug sensitivity assays comparing control Clone 1, miR-181a KO C7, and miR-181a KO C16 cell lines treated with different concentrations of olaparib (One-way ANOVA and Sidak’s multiple comparisons test). *p < 0.05, **p < 0.01, ***p < 0.001. **D** Representative images of miR-181a ISH in pre-treatment (Pre-Tx) and post-treatment (Post-Tx) paired tissues from OvCa patients. Images of negative control probes (Negative CTRL), positive control probes (Positive CTRL), Pre-Tx, and Post-Tx paired FFPE tissues from OvCa patient #1 (P01). **E** Quantification of miR-181a levels (dots/nuclei) in Pre-Tx and Post-Tx paired tissues from OvCa patients (Student’s t-test). *p < 0.05, **p < 0.01, ***p < 0.001. Cell viability assays were performed in triplicates. **F**–**I** Correlation between miR-181a levels and cisplatin (**F**), rucaparib (**G**), olaparib (**H**), talazoparib (**I**) drug activity in the BC and OvCa cell lines obtained from CCLE and GDSC BRCA datasets (Pearson’s correlation coefficient)

Low miR-181a levels are associated with a better cisplatin response [16]. To assess whether this association was also observed with PARPi, two isogenic OvCa cell line pairs (OV81.2 and OV231) were analyzed. The two OvCa cell line pairs were generated through platinum-drug selection and became cisplatin and PARPi resistant [25, 26]. We found that the miR-181a levels were significantly increased in cisplatin- and PARPi-resistance cell lines compared to corresponding parental cell lines (Fig. 7A). To determine if miR-181a can confer cross-resistance in OvCa cell lines, we developed two miR-181a knock-out (KO) CRISPR-engineered clones using patient-derived OvCa cell lines [26], that were sequenced and further validated by RT-qPCR (Fig. 7B). Consistent with the previous observations in TNBC cell lines, miR-181a KO increased the sensitivity to olaparib in patient-derived OvCa cell lines (Fig. 7C). To determine if the miR-181a levels have clinical significance, we selected a small cohort of OvCa patients who had surgery before adjuvant therapy (carboplatin-paclitaxel) and debulking surgery after patients failed adjuvant therapy. The miR-181a levels were assessed by ISH assays, using paired FFPE tumor tissues from OvCa patients. Tumor tissue samples resected after treatment failure (Post-Tx) had significantly higher miR-181a levels compared to pre-treatment (Pre-Tx) tumor tissues (Fig. 7D, E).

To further support the role of miR-181a in promoting PARPi and platinum-based drug resistance, we performed *in-silico* analysis using CCLE and GDSC databases using BC and OvCa cell lines. The results demonstrated that miR-181a levels significantly negative correlated with the sensitivity to cisplatin and PARPi (rucaparib, olaparib, and talazoparib) in both BC and OvCa cell lines (Fig. 7F–I and Additional file 1: Fig. S5A–H). In summary, the increased miR-181a levels in post-treatment samples may represent a mechanism of intrinsic resistance in OvCa tumors, nonetheless, patients who failed adjuvant therapy showed increased miR-181a levels in pre-treatment samples. The miR-181a plays an important role in resistance to platinum-based and PARPi in BC and OvCa.

## Discussion

The early detection of drug resistance as well as the understanding of the intrinsic mechanisms that induced PARPi resistance are important for both TNBC and OvCa patients. Thus, the prospects for developing effective strategies for the early detection of PARPi resistance are a growing field. Previous studies have shown that germline and somatic *BRCA* mt are well-defined biomarkers for PARPi response in several cancer types including BC and OvCa [2, 3]. Alterations in genes that are part of the HR pathway have also been associated with improved responses to PARPi [27–29]. A recent post-hoc exploratory biomarker analysis for the patients, who were enrolled in the ARIEL2 trial a single-arm open-label phase 2 study of the rucaparib in relapsed high-grade OvCa, demonstrated that *RAD51C*/*D* mutations (mt) and *BRCA1* promoter methylation predicted response to rucaparib [30].

Tumors that are sensitive to platinum-based drugs are also sensitive to PARPi treatment [31, 32]. Our group showed STING expression/activation significantly increases platinum-based drug sensitivity in TNBC and that the proteasomal shuttle factor UBQLN4 promotes STING degradation [12]. Consequently, STING downregulation mediated by UBQLN4 leads to platinum resistance in TNBC [12]. The present study showed that STING downregulation led to PARPi resistance through decreased proinflammatory cytokine levels and IFNG responses in TNBC. Furthermore, STING levels and IFNG responses predicted treatment responses to neoadjuvant therapy (DOP) in high-risk HER2 negative BC. Thus, suggesting that STING represents a potential predictor PARPi and platinum-based drug cross-resistance.

The present study offers additional insight into miR-181a regulatory functions controlling STING mRNA and protein levels. MiR-181a targets STING and promotes metastasis as well as recurrence in advanced stage HGSOC [33]. Our study demonstrates that miR-181a-mediated STING downregulation leads to decreased downstream proinflammatory cytokines and IFNG responses in TNBC. Both STING and miR-181a may serve as promising biomarkers for PARPi sensitivity.

Several studies have shown that the activation of STING and the stimulation of type I IFN production are critical for anticancer immune responses. Activation of STING signaling induces the production of type I IFN, which plays critical roles in activating both innate and adaptive immune responses [34]. A study showed that PARPi activates the STING pathway and stimulates the production of type I IFN to induce antitumor immunity [35]. Thus, activation of the STING pathway would facilitate cancer cells to death.

Several clinical trials have been conducted to test the efficacy of PARPi plus immune checkpoint inhibitors (ICI) in ovarian, breast, prostate, lung, bladder, gastric cancers, and other solid tumors [36]. MiR-181a may serve as a biomarker not only for PARPi monotherapy, but also in PARPi plus ICI combination therapy since STING activation will be reduced in cancer cells with high miR-181a levels as shown in our results. Further clinical studies may be needed to verify the impact of miR-181a as a biomarker during ICI.

Recently, EVs have attracted extensive attention for their role in drug resistance [37, 38]. The functional proteins or non-coding RNAs contained in EV released from tumor and stromal cells mediate drug resistance by regulating drug efflux and metabolism, pro-survival signaling, epithelial-mesenchymal transition, stem-like property, and tumor microenvironmental remodeling [37]. Previous reports showed that miR-181a in EV promotes the development of early-stage myeloid-derived suppressor cells by interfering with protein inhibitor of activated STAT3 (*PIAS3*) [39], which targets mixed-lineage leukemia 3 (MLL3), and therefore induced angiogenesis and tumor growth in papillary thyroid cancer cells [40]. This study reveals for the first time, a crucial role of EVs-derived from OlaR TNBC cells that induced PARPi resistance by horizontal transfer of miR-181a in vitro. Nonetheless, we proposed that EVs that are released by tumor cells can affect tumor-adjacent normal cells by transferring high levels of miR-181a. EVs-containing miR-181a may become a useful biomarker to predict PARPi treatment responses. Future clinical studies are needed to standardize EVs isolation and miR extraction from EVs to verify the utility of EV-derived miR-181a as a marker predicting PARPi sensitivity.

## Conclusions

**Fig. 8:**
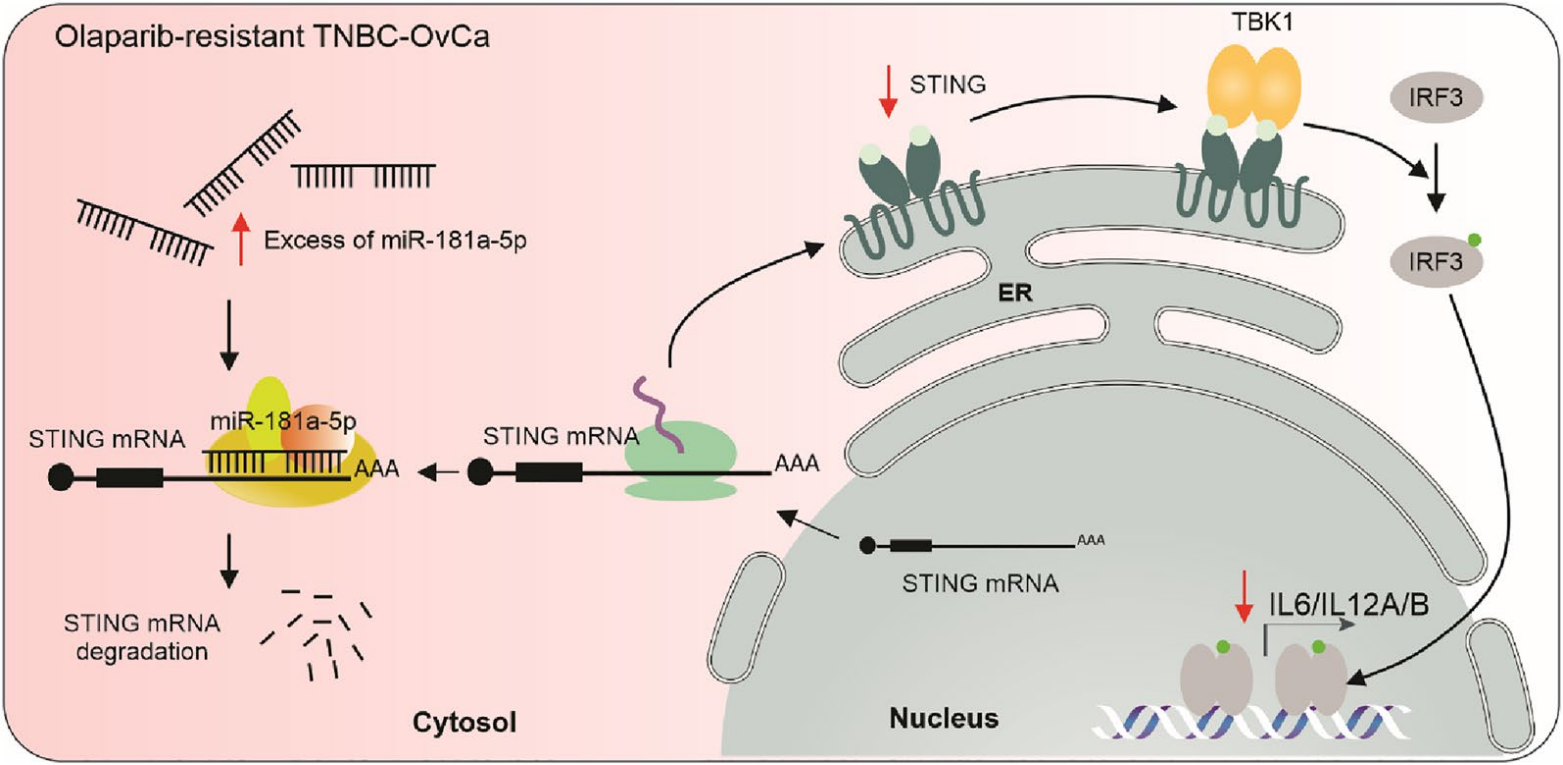
Schematic of miR-181a-5p mediated regulation on STING. The upregulation of miR-181a-5p in olaparib-resistant cells increases STING degradation by targeting STING mRNA. Consequently, the STING downstream pathways is downregulated

TNBC cell lines resistant to PARPi showed enhanced levels of miR-181a (Fig. 8). MiR-181a targets STING and consequently, miR-181a reduced the downstream proinflammatory cytokines. High miR-181a levels were also associated with low IFNG responses in TNBC. High miR-181a/low STING/low IFNG responses were associated with poor responses to neoadjuvant modalities (including PARPi). Furthermore, the horizontal transfer of miR-181a in TNBC cells-derived EVs induced PARPi resistance in sensitive TNBC cell lines. This study unravels a new role for miR-181a in promoting PARPi resistance and suggests that miR-181a and STING are promising markers associated with PARPi responses in TNBC and OvCa.

### Supplementary Information

**Additional file 1: **Material and Methods. **Figure S1. **TNBC olaparib-resistant cell lines and miR-181a overexpressing cell lines showed cross-resistance to cisplatin. **A** Schematic representation of the seed sequences for miR-181a-5p, miR-181b-5p, miR-181c-5p, and miR-181d-5p in the 3′UTR of TMEM173 gene. **B**–**C**. Drug sensitivity assays comparing parental and olaparib-resistant (OlaR) HCC1937 cell line treated with different concentrations of olaparib (B) or cisplatin (C) (Two-way ANOVA and Sidak’s multiple comparisons test). **D** Cell viability assays comparing parental and OlaR HCC1937 cell line (Two-way ANOVA and Sidak’s multiple comparisons test). **E **Representative images for GFP positive cells with miR-181a-OV in MDA-MB-436, HCC1395, and HCC1937 cell lines using fluorescence microscopy. Scale bars: 100 µm. **F**–**H** Cell viability assays comparing miR-181a-OV and empty vector (CTRL) in MDA-MB-436 (E), HCC1395 (F), and HCC1937 (G) cell lines (two-way ANOVA and Sidak’s multiple comparisons test). **I** Cell viability assays comparing miR-181a-OV and empty vector (CTRL) HCC1937 cell lines treated with different concentrations of cisplatin (two-way ANOVA and Sidak’s multiple comparisons test). Cell viability assays were performed in triplicates. **Figure S2. **STING overexpression or downregulation does not affect cell proliferation. **A** Representative images for STING-OV (GFP) in MDA-MB-436, HCC1395, and HCC1937 cell lines using fluorescence microscopy. Scale bars: 100 µm. **B**–**D** Cell viability assays comparing STING-OV and empty vector (CTRL) in MDA-MB-436 (**B**), HCC1395 (**C**), and HCC1937 (**D**) cell lines (two-way ANOVA and Sidak’s multiple comparisons test). **E**–**G** Cell viability assays comparing si-STING and si-CTRL in MDA-MB-436 (**E**), HCC1395 (**F**), and HCC1937 (**G**) (two-way ANOVA and Sidak’s multiple comparisons test). **Figure S3. **STING is downregulated in TNBC and relates to outcomes. **A*** STING *mRNA levels in normal breast (Normal) and primary BC (Primary) tissues in the TCGA and GTEx databases (Student’s t-test). **B** *STING* mRNA levels in tissues from tumor-adjacent normal breast (Adj. Normal), Luminal (Lum), Luminal-HER2 (Lum-HER2), HER2, and TNBC in the TCGA BRCA dataset (One-way ANOVA and Tukey’s multiple comparisons test). **C** *STING* mRNA levels in tissues from the tumor-adjacent normal breast (Adj. Normal), Normal-like (Norm-like), Luminal-A (LumA), Luminal-B (LumB), HER2-enriched, and basal-like breast cancer (BLBC) in the TCGA BRCA dataset (One-way ANOVA and Tukey’s multiple comparisons test). **D**–**F** Survival analysis of RFS **(D**), OS (**E**), and DMFS (**F**) for patients with TNBC in the TCGA, GEO, and EGA databases combined (Log-rank test).** G**–**I** Survival analysis of RFS **(G**), OS (**H**), and DMFS (**I**) for patients with BLBC in the TCGA, GEO, and EGA database combined (Log-rank test). *p<0.05, **p <0.01, ***p <0.001. **Figure S4. **Analysis of the mRNA levels of the downstream components of the STING pathways. **A**–**E** Quantification by RT-qPCR of *STING *(A), *IL6* (B), *IFNB* (C), *IL12A* (D), and *IL12B* (E) mRNA levels in empty vector (CTRL) and STING-OV HCC1395 cell line (Student’s t-test). **F**–**J** Quantification by RT-qPCR of *STING *(F), *IL6* (G), *IFNB* (H), *IL12A* (I), and *IL12B *(J) mRNA levels in empty vector (CTRL) and STING-OV MDA-MB-436 cell line (Student’s t-test). **K**–**O** Quantification by RT-qPCR of *STING *(K),* IL6 *(L),* IFNB *(M),* IL12A* (N), and* IL12B *(O) mRNA levels in miR-181a-OV and CTRL in HCC1395 cell line (Student’s t-test). **P**-**T** Quantification by RT-qPCR of *STING *(P), *IL6 *(Q),* IFNB *(R), *IL12A* (S), and *IL12B *(T) mRNA levels in miR-181a-OV and empty vector (CTRL) in MDA-MB-436 cell line (Student’s t-test). *p<0.05, **p <0.01, ***p <0.001. **Figure S5. **Correlation analysis between miR-181a levels and drug activities. **A**–**D** Correlation between miR-181a levels and cisplatin (**A**), rucaparib (**B**), olaparib (**C**), talazoparib (**D**) drug activity in the BC cell lines obtained from CCLE and GDSC BRCA datasets (Pearson’s correlation coefficient). **F**–**I** Correlation between miR-181a levels and cisplatin (**F**), rucaparib (**G**), olaparib (**H**), talazoparib (**I**) drug activity in the OvCa cell lines obtained from CCLE and GDSC BRCA datasets (Pearson’s correlation coefficient). **Figure S6. **Uncropped western blotting images. **A-C **Uncropped western blotting images for Figures 3**D** (A), **H** (B), and **I** (C) are shown. **Figure S7. **Uncropped western blotting images. **A**–**D**. Uncropped western blotting images for Figures 3**J** (**A**), **K** (B), 4**A** (**C**), and **E** (**D**) are shown. **Figure S8. **Uncropped western blotting images. **A** Uncropped western blotting images for Figure 6**A** (A) and **G** (B) are shown.

**Additional file 2: Table S1.** Reagents and resources utilized in the study.

## Data Availability

All the in-silico data analyzed is publicly available. Datasets from The Cancer Genome Atlas (TCGA) breast cancer (BRCA) [41] and Genotype-Tissue Expression (GTEx) Breast-Mammary Tissue [42] were downloaded through the University of California Santa Cruz (UCSC) Xena [43]. Datasets from The Cancer Cell Line Encyclopedia (CCLE) [44] and Genomics of Drug Sensitivity in Cancer (GDSC) [45] were downloaded through CellMiner Cross-Database (CellMinerCDB) Version 1.4 [46]. Survival analyses were performed with the Kaplan–Meier Plotter [47] using the TCGA, Gene Omnibus Expression (GEO) [48], and European Genome-Phenome Archive (EGA) [49] databases either alone or combined.

## Abbreviations

ACE: Alternating current electrokinetic platform
BC: Breast cancer
BLBC: Basal-like breast cancer
cGAMP: Cyclic guanosine monophosphate-adenosine monophosphate
CCLE: Cancer cell line encyclopedia
CR: Complete response
DE: Differentially expressed
DUC: Differential ultracentrifugation
EVs: Extracellular vesicles
FDR: False rate discovery
FFPE: Formalin-fixed paraffin-embedded
FTSEC: Fallopian tube secretory epithelial cells
FC: Fold-change
FL-NTA: Fluorescent nanoparticles tracking analysis
gBRCA mt: Germline *BRCA*-mutated
GDSC: Genomics of drug sensitivity in cancer
GEE: Gene omnibus expression
GTEx: Genotype-tissue expression
HGSOC: High-grade serous ovarian cancer
HR: Homologous recombination
HTG miR WTA: EdgeSeq miRNA whole transcriptome assay
ICI: Immune checkpoint inhibitors
IDC: Invasive ductal carcinoma
IFNB: Interferon beta
IFNG: Interferon gamma
IHC: Immunohistochemistry
IL6: Interleukin-6
IL12A: Interleukin-12A
IL12B: Interleukin-12B
ISH: *in-situ* hybridization
IRF3: Interferon regulatory factor 3
KO: Knock-out
mt: mutation
NGS: Next-generation sequencing
Non-CR: Non-complete response
NTA: Nanoparticle tracking analysis
OlaR: Olaparib-resistant
OvCa: Ovarian cancer
OS: Overall survival
PARPi: Poly (ADP-ribose) polymerase inhibitors.
Pre-Tx: Pre-treatment
Post-Tx: Post-treatment
RT-qPCR: Reverse transcription-quantitative polymerase chain reaction
RFS: Recurrence-free survival.
STING: Stimulator of interferon genes
TBK1: TANK-binding kinase 1
TCGA: The Cancer Genome Atlas
TMA: Tissue microarray
TNBC: Triple-negative breast cancer
