# Supplementary material for: MiR-181a targets STING to drive PARP inhibitor resistance in BRCA- mutated triple-negative breast cancer and ovarian cancer
Source: Cell Biosci. 2023 Nov 6;13:200. doi: 10.1186/s13578-023-01151-y (PMC10626784; doi:10.1186/s13578-023-01151-y)
Supplement: Supplementary file 1 — Additional file 1: Material and Methods. Figure S1. TNBC olaparib-resistant cell lines and miR-181a overexpressing cell lines showed cross-resistance to cisplatin. A Schematic representation of the seed sequences for miR-181a-5p, miR-181b-5p, miR-181c-5p, and miR-181d-5p in the 3′UTR of TMEM173 gene. B–C. Drug sensitivity assays comparing parental and olaparib-resistant (OlaR) HCC1937 cell line treated with different concentrations of olaparib (B) or cisplatin (C) (Two-way ANOVA and Sidak’s multiple comparisons test). D Cell viability assays comparing parental and OlaR HCC1937 cell line (Two-way ANOVA and Sidak’s multiple comparisons test). E Representative images for GFP positive cells with miR-181a-OV in MDA-MB-436, HCC1395, and HCC1937 cell lines using fluorescence microscopy. Scale bars: 100 µm. F–H Cell viability assays comparing miR-181a-OV and empty vector (CTRL) in MDA-MB-436 (E), HCC1395 (F), and HCC1937 (G) cell lines (two-way ANOVA and Sidak’s multiple comparisons test). I Cell viability assays comparing miR-181a-OV and empty vector (CTRL) HCC1937 cell lines treated with different concentrations of cisplatin (two-way ANOVA and Sidak’s multiple comparisons test). Cell viability assays were performed in triplicates. Figure S2. STING overexpression or downregulation does not affect cell proliferation. A Representative images for STING-OV (GFP) in MDA-MB-436, HCC1395, and HCC1937 cell lines using fluorescence microscopy. Scale bars: 100 µm. B–D Cell viability assays comparing STING-OV and empty vector (CTRL) in MDA-MB-436 (B), HCC1395 (C), and HCC1937 (D) cell lines (two-way ANOVA and Sidak’s multiple comparisons test). E–G Cell viability assays comparing si-STING and si-CTRL in MDA-MB-436 (E), HCC1395 (F), and HCC1937 (G) (two-way ANOVA and Sidak’s multiple comparisons test). Figure S3. STING is downregulated in TNBC and relates to outcomes. A STING mRNA levels in normal breast (Normal) and primary BC (Primary) tissues in the TCGA and GTEx databases (Student [file 13578_2023_1151_MOESM1_ESM.docx]

**MiR-181a Targets STING to Drive PARP Inhibitor Resistance in *BRCA*-mutated Triple-Negative Breast Cancer and Ovarian Cancer**

Matias A. Bustos^1^, Takamichi Yokoe^1^, Yoshiaki Shoji^1^, Yuta Kobayashi^1^, Shodai Mizuno^1^, Tomohiro Murakami^1^, Xiaoqing Zhang^1^, Sreeja C. P. Sekhar^5,6^, SooMin Kim^2^, Suyeon Ryu^2^, Matthew Knarr^3^, Steven A. Vasilev^4^, Analisa DiFeo^5,6^, Ronny Drapkin^3^, and Dave S. B. Hoon^1,2^

^1^Department of Translational Molecular Medicine, Saint John’s Cancer Institute (SJCI) at Providence Saint John’s Health Center (SJHC), Santa Monica, CA, 90404, USA.

^2^Department of Genome Sequencing, SJCI at Providence SJHC, Santa Monica, CA, 90404, USA.

^3^Department of Obstetrics and Gynecology, Perelman School of Medicine, Penn Ovarian Cancer Research Center, University of Pennsylvania, Pennsylvania, PA, 19104, USA.

^4^Department of Gynecologic Oncology Research, SJCI at SJHC, Santa Monica, CA, 90404, USA.

^5^Department of Obstetrics & Gynecology, University Michigan, Ann Arbor, MI, 48109, USA.

^6^Department of Pathology, Rogel Cancer Center, University Michigan, Ann Arbor, MI, 48109, USA.

**Supplementary Material and Methods**

1. **Establishment of TNBC olaparib -resistant cell lines**

For the establishment of the PARPi olaparib-resistant (OlaR) TNBC cell line, the HCC1937 and HCC1395 cell lines were cultured in medium containing 1 μM of Olaparib (#S1060, Selleck Chemicals, Houston, TX) supplemented medium for 14 days. Afterward, the medium was replaced, and the cell lines were cultured until the resistant clones proliferated; the treatment was repeated with 2, 5, 10, 15, and then 30 μM respectively, of Olaparib supplemented medium. Cell lines were recovered in an Olaparib-free medium and used for further assays.

The establishment of the cisplatin- and olaparib-resistant OvCa cell line OV81.2 and OV231 were derived from OvCa patients as previously described (1,2).

1. **miRNA hairpin inhibitor assay**

TNBC cell lines were transfected with miRIDIAN microRNA Hairpin Inhibitor (Cat# IH-300552-07-0005, Horizon, Colorado) at 10 nM, 25 nM, or 50 nM concentrations to target miR-181a-5p; or with miRIDIAN microRNA Hairpin Inhibitor Negative Control #1 (Cat# CN-001000-01-05, Horizon) at 25 nM or 50 nM concentrations following manufacturers’ recommendations.

1. **RNA isolation from EV**

Total RNA isolation from isolated extracellular vesicles (EV) was performed with Plasma/Serum RNA purification mini kit (#55000, Norgen Biotek, Thorold, Ontario, Canada) following the manufacturer’s recommendation as previously described (3) at the Extracellular Vesicle Core, Children’s Hospital Los Angeles, Los Angeles, CA. Complementary DNA (cDNA) synthesis was performed for 250 ng of total RNA using qScript™ microRNA cDNA Synthesis Kit (#95107-025, Quanta Biosciences, Beverly, MA) according to the manufacturer’s instructions.

1. **CRISPR Cas9 cell line generation**

The miR-181a knockout (KO) and control CRISPR clones were generated by the Vector Core at the University of Michigan. Single cells were seeded into a 96-well plate and positive clones were verified by sequencing. Clones C1 was used a control and clones C7 and C16 were KO for miR-181a. The single guide RNAs (sgRNA) sequences targeting hsa-miR-181a-5p were obtained are described in **Table S1**.

1. **Isolation of TNBC cell lines-derived EVs**

EVs released by TNBC cell lines were isolated as previously described (4). Briefly, cells were plated in two 150 mm dishes and were cultured until 80% confluent. Cells were rinsed twice with sterile physiological phosphate buffered saline (PBS) pH7.4 and incubated with 20 mL serum-free culture media for 12 hours. The conditioned medium (CM) was collected and centrifuged at 1,000 x g for 5 minutes at 4°C to remove intact cells and debris. The CMs were centrifuged at 10,000 x g for 30 minutes at 4°C, followed by ultracentrifugation at 100,000 x g for 90 minutes at 4°C to pellet EVs with Optima MAX-TL (Beckman Coulter, Brea, CA) using TLA-100.3 fixed-angle rotor (k-factor: 14, Beckman Coulter) and thick wall polycarbonate tubes (#349622, Beckman Coulter). The supernatants were carefully removed, and crude EV-containing pellets were resuspended in 500 µL of ice-cold PBS and pooled. The second ultracentrifugation was performed at 100,00 x g for 90 minutes at 4°C, and the EV-containing pellet was resuspended in 100 µL of PBS pH 7.4.

1. **Characterization of EVs**

Briefly, isolated EV samples were analyzed using a Nanosight NS300 instrument (Malvern Panalytical, Malvern, UK) equipped with a green 532 nm laser and an sCMOS camera (EV core lab, Children’s Hospital Los Angeles). The samples were diluted (500 to 5,000-fold dilutions) to obtain the optimal detection concentration of 108 particles/ml. The data were analyzed using NTA software 4.3 with detection threshold 7 and adjusted by the dilution factor to compare the homogeneity, the size, and the number of particles. For fluorescent NTA, isolated EVs were mixed with a 1:1,000 dilution of the CellMask Orange Plasma Membrane Stain (Invitrogen, ThermoFisher) at a 1:1 ratio, incubated in the dark for 10 minutes, and analyzed on the nanosight equipped with a 565 long pass filter and with camera settings of 16 and manual histogram limits of 200/1,800. Fluorescent-NTA using CellMask Orange Plasma Membrane Stain was performed to detect only the membrane-enclosed particles as previously described (5). Western blotting was performed to confirm the presence of known EV surface markers (6). Alternating current electrokinetic platform (ACE, ExoVerita, Biological Dynamics, San Diego, CA) was utilized to visualize CD9 positive EVs as previously described (7,8). ACE images were taken using the ECHO Revolve microscope and the ECHO Pro App. Ab dilutions for western blotting and ACE are shown in **Table S1**.

1. **Analysis of public datasets: I-SPY2**

RNA sequencing (RNA-seq, GSE173839) data for FFPE tissues from pretreatment biopsies and clinical information for a subset of patients who were enrolled in the I-SPY2 trial were downloaded from GEO (9). I-SPY2 is a randomized phase II trial to investigate the efficacy of neoadjuvant PD-L1 inhibitor (Durvalumab) and PARPi (Olaparib) added to standard paclitaxel therapy (Durvalumab/Olaparib/Paclitaxel, or DOP) for high-risk HER2 negative stage II/III BC.

1. **Biostatistics and bioinformatics analysis**

Patients were divided into two groups using the best cut-off value in each analysis by Kaplan-Meier Plotter software. IFNG response scores were calculated by the average gene expression of the six IFNG gene signatures (indoleamine 2,3-dioxygenase 1 [*IDO1*], C-X-C motif chemokine ligand 9/10 [*CXCL9*/*CXCL10*], major histocompatibility complex, class II, DR Alpha [*HLA-DRA*], signal transducer and activator of transcription 1 [*STAT1*], and *IFNG*) as previously described (10).

**Supplemental data**


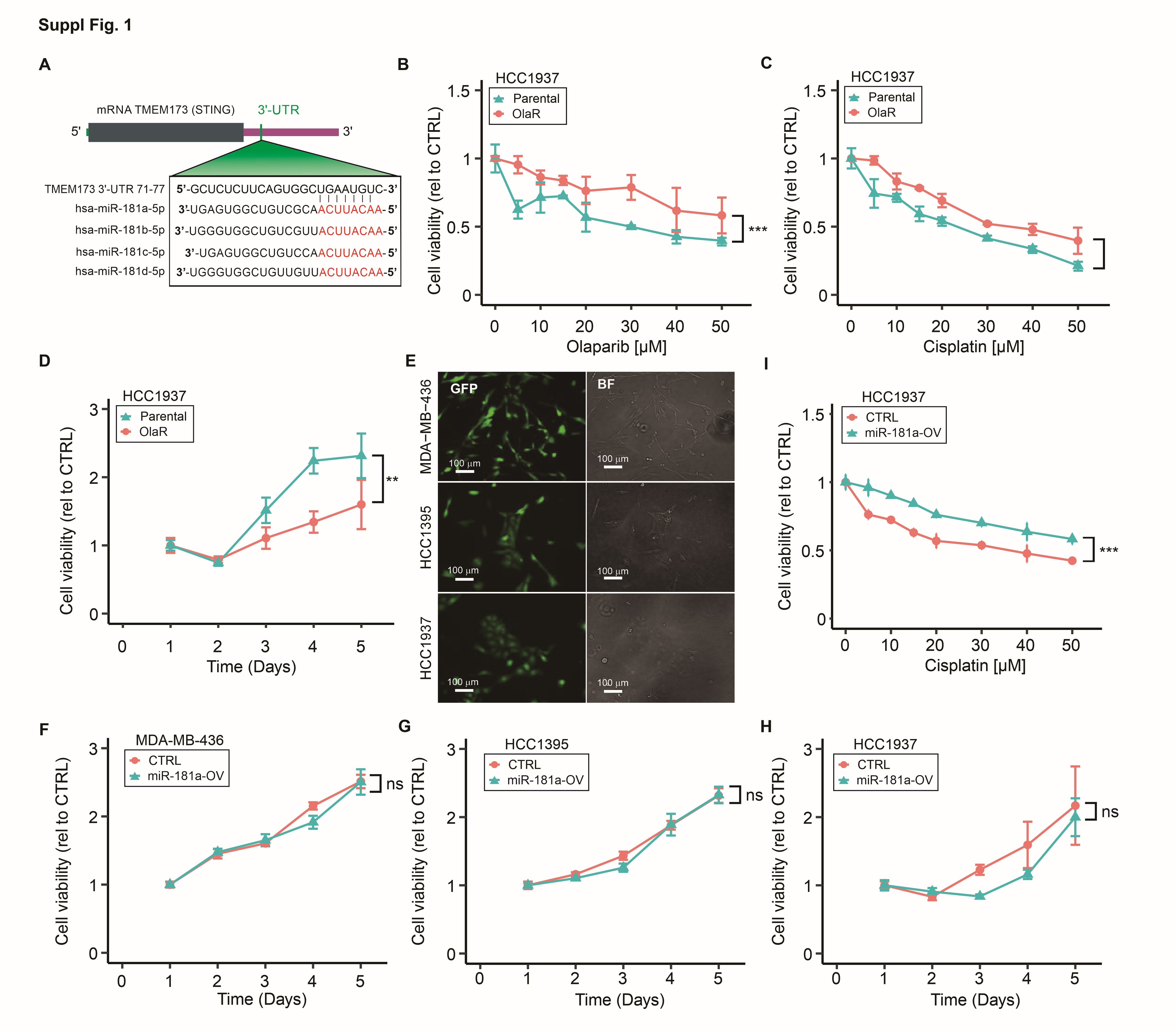


**Figure S1. TNBC olaparib-resistant cell lines and miR-181a overexpressing cell lines showed cross-resistance to cisplatin. A.** Schematic representation of the seed sequences for miR-181a-5p, miR-181b-5p, miR-181c-5p, and miR-181d-5p in the 3’UTR of TMEM173 gene. **B-C.** Drug sensitivity assays comparing parental and olaparib-resistant (OlaR) HCC1937 cell line treated with different concentrations of olaparib (B) or cisplatin (C) (Two-way ANOVA and Sidak’s multiple comparisons test). **D.** Cell viability assays comparing parental and OlaR HCC1937 cell line (Two-way ANOVA and Sidak’s multiple comparisons test). **E.** Representative images for GFP positive cells with miR-181a-OV in MDA-MB-436, HCC1395, and HCC1937 cell lines using fluorescence microscopy. Scale bars: 100 µm. **F-H.** Cell viability assays comparing miR-181a-OV and empty vector (CTRL) in MDA-MB-436 (E), HCC1395 (F), and HCC1937 (G) cell lines (two-way ANOVA and Sidak’s multiple comparisons test). **I.** Cell viability assays comparing miR-181a-OV and empty vector (CTRL) HCC1937 cell lines treated with different concentrations of cisplatin (two-way ANOVA and Sidak’s multiple comparisons test). Cell viability assays were performed in triplicates.


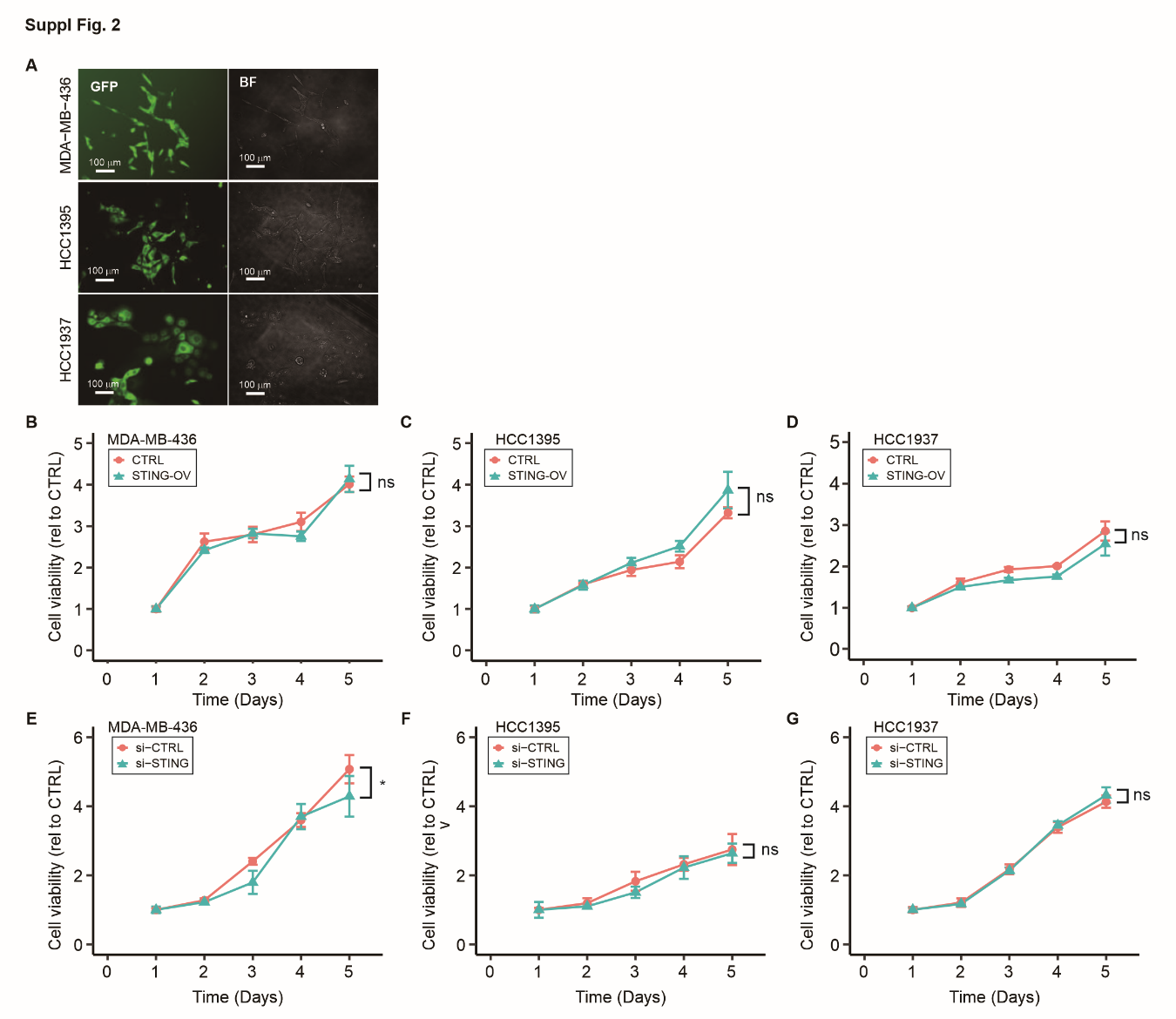


**Figure S2. STING overexpression or downregulation does not affect cell proliferation. A.** Representative images for STING-OV (GFP) in MDA-MB-436, HCC1395, and HCC1937 cell lines using fluorescence microscopy. Scale bars: 100 µm. **B-D.** Cell viability assays comparing STING-OV and empty vector (CTRL) in MDA-MB-436 (**B**), HCC1395 (**C**), and HCC1937 (**D**) cell lines (two-way ANOVA and Sidak’s multiple comparisons test). **E-G.** Cell viability assays comparing si-STING and si-CTRL in MDA-MB-436 (**E**), HCC1395 (**F**), and HCC1937 (**G**) (two-way ANOVA and Sidak’s multiple comparisons test).


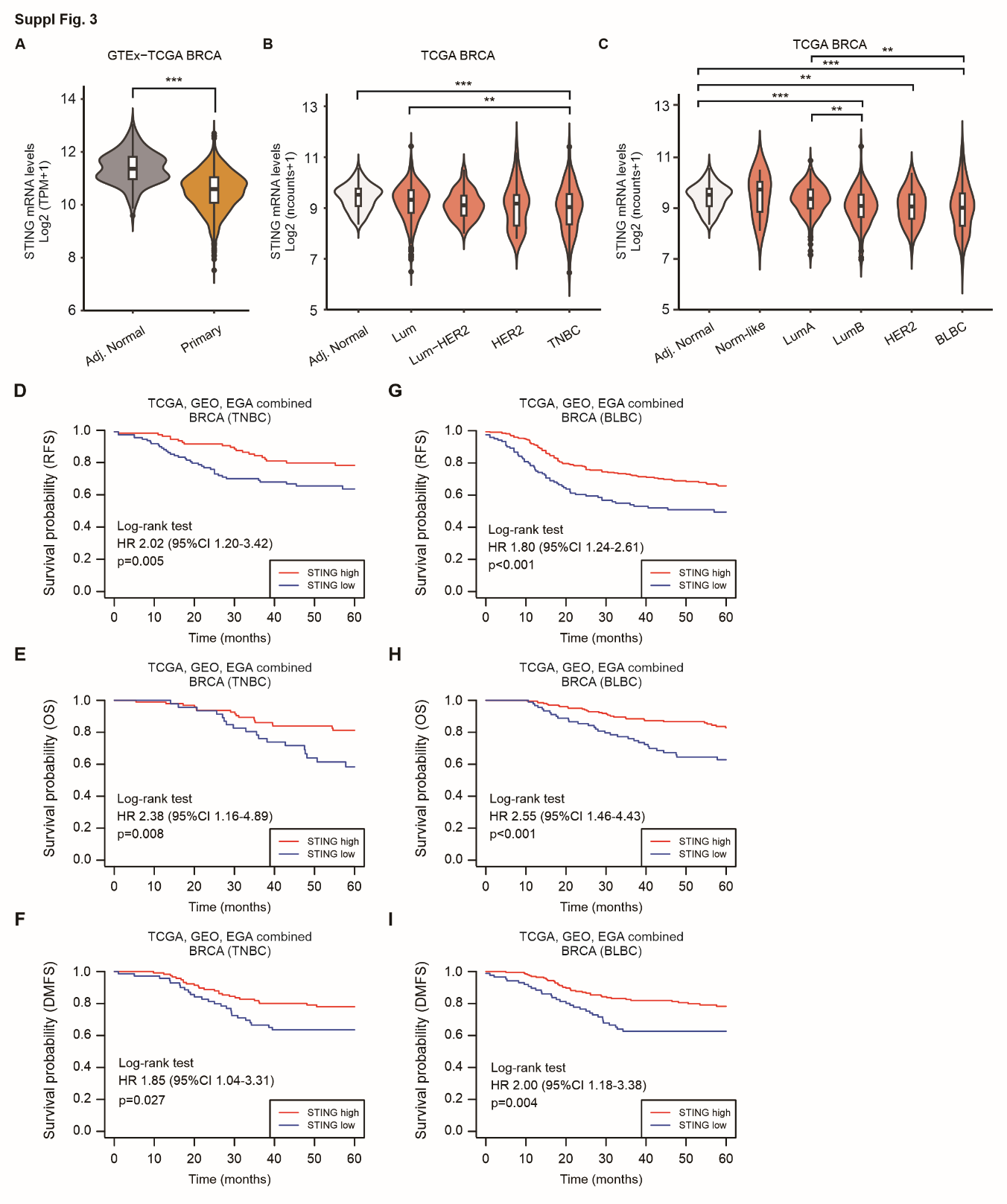


**Figure S3. STING is downregulated in TNBC and relates to outcomes. A.** *STING* mRNA levels in normal breast (Normal) and primary BC (Primary) tissues in the TCGA and GTEx databases (Student’s t-test). **B.** *STING* mRNA levels in tissues from tumor-adjacent normal breast (Adj. Normal), Luminal (Lum), Luminal-HER2 (Lum-HER2), HER2, and TNBC in the TCGA BRCA dataset (One-way ANOVA and Tukey’s multiple comparisons test). **C.** *STING* mRNA levels in tissues from the tumor-adjacent normal breast (Adj. Normal), Normal-like (Norm-like), Luminal-A (LumA), Luminal-B (LumB), HER2-enriched, and basal-like breast cancer (BLBC) in the TCGA BRCA dataset (One-way ANOVA and Tukey’s multiple comparisons test). **D-F**. Survival analysis of RFS **(D**), OS (**E**), and DMFS (**F**) for patients with TNBC in the TCGA, GEO, and EGA databases combined (Log-rank test). **G-I**. Survival analysis of RFS **(G**), OS (**H**), and DMFS (**I**) for patients with BLBC in the TCGA, GEO, and EGA database combined (Log-rank test). * *p*<0.05, ** *p* <0.01, *** *p* <0.001.


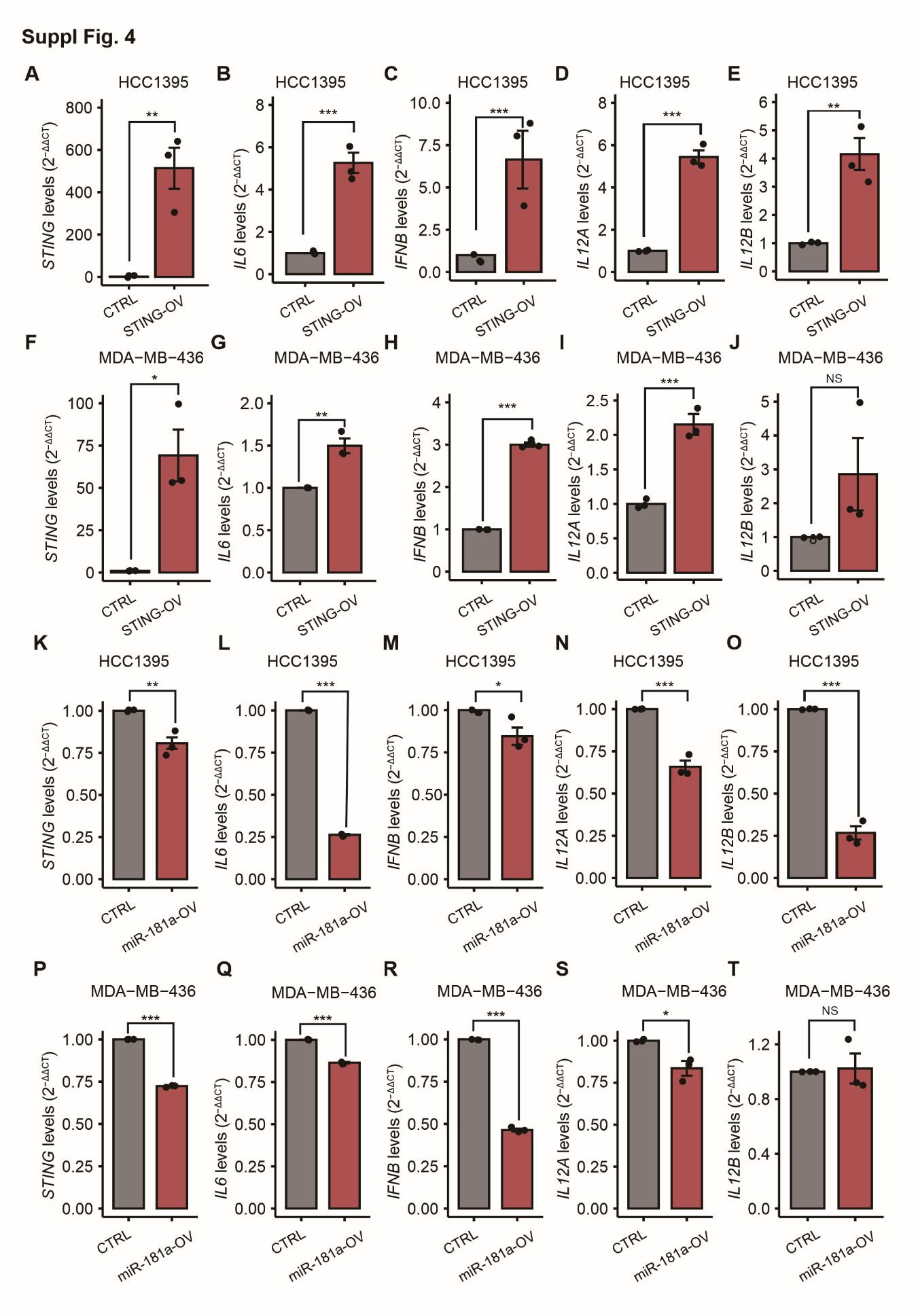


**Figure S4. Analysis of the mRNA levels of the downstream components of the STING pathways.** **A-E.** Quantification by RT-qPCR of *STING* (A), *IL6* (B), *IFNB* (C), *IL12A* (D), and *IL12B* (E) mRNA levels in empty vector (CTRL) and STING-OV HCC1395 cell line (Student’s t-test). **F-J.** Quantification by RT-qPCR of *STING* (F), *IL6* (G), *IFNB* (H), *IL12A* (I), and *IL12B* (J) mRNA levels in empty vector (CTRL) and STING-OV MDA-MB-436 cell line (Student’s t-test). **K-O.** Quantification by RT-qPCR of *STING* (K), *IL6* (L), *IFNB* (M), *IL12A* (N), and *IL12B* (O) mRNA levels in miR-181a-OV and CTRL in HCC1395 cell line (Student’s t-test). **P-T.** Quantification by RT-qPCR of *STING* (P), *IL6* (Q), *IFNB* (R), *IL12A* (S), and *IL12B* (T) mRNA levels in miR-181a-OV and empty vector (CTRL) in MDA-MB-436 cell line (Student’s t-test). * *p*<0.05, ** *p* <0.01, *** *p* <0.001.


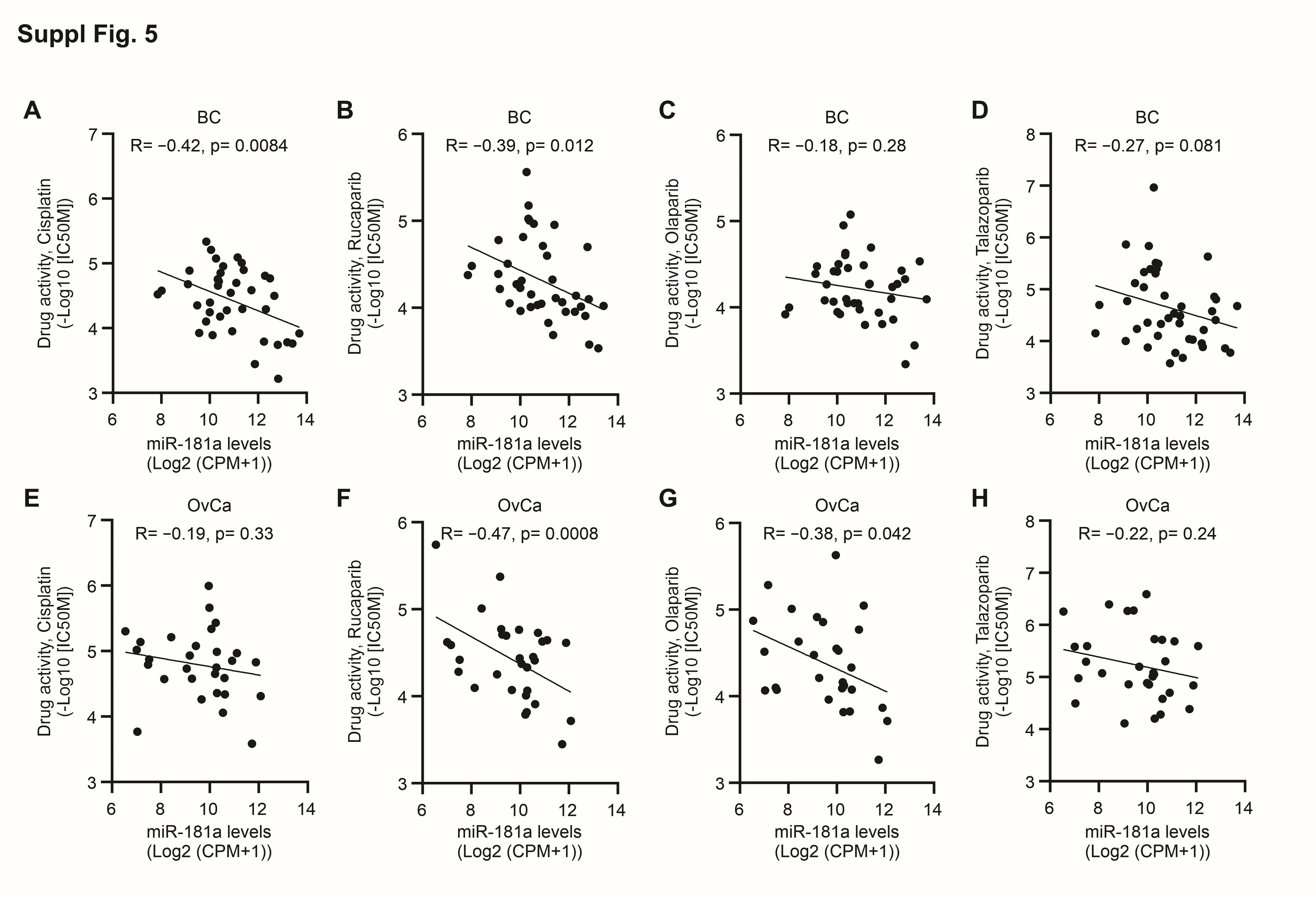


**Figure S5. Correlation analysis between miR-181a levels and drug activities. A****-D.** Correlation between miR-181a levels and cisplatin (**A**), rucaparib (**B**), olaparib (**C**), talazoparib (**D**) drug activity in the BC cell lines obtained from CCLE and GDSC BRCA datasets (Pearson’s correlation coefficient). **F-I.** Correlation between miR-181a levels and cisplatin (**F**), rucaparib (**G**), olaparib (**H**), talazoparib (**I**) drug activity in the OvCa cell lines obtained from CCLE and GDSC BRCA datasets (Pearson’s correlation coefficient).


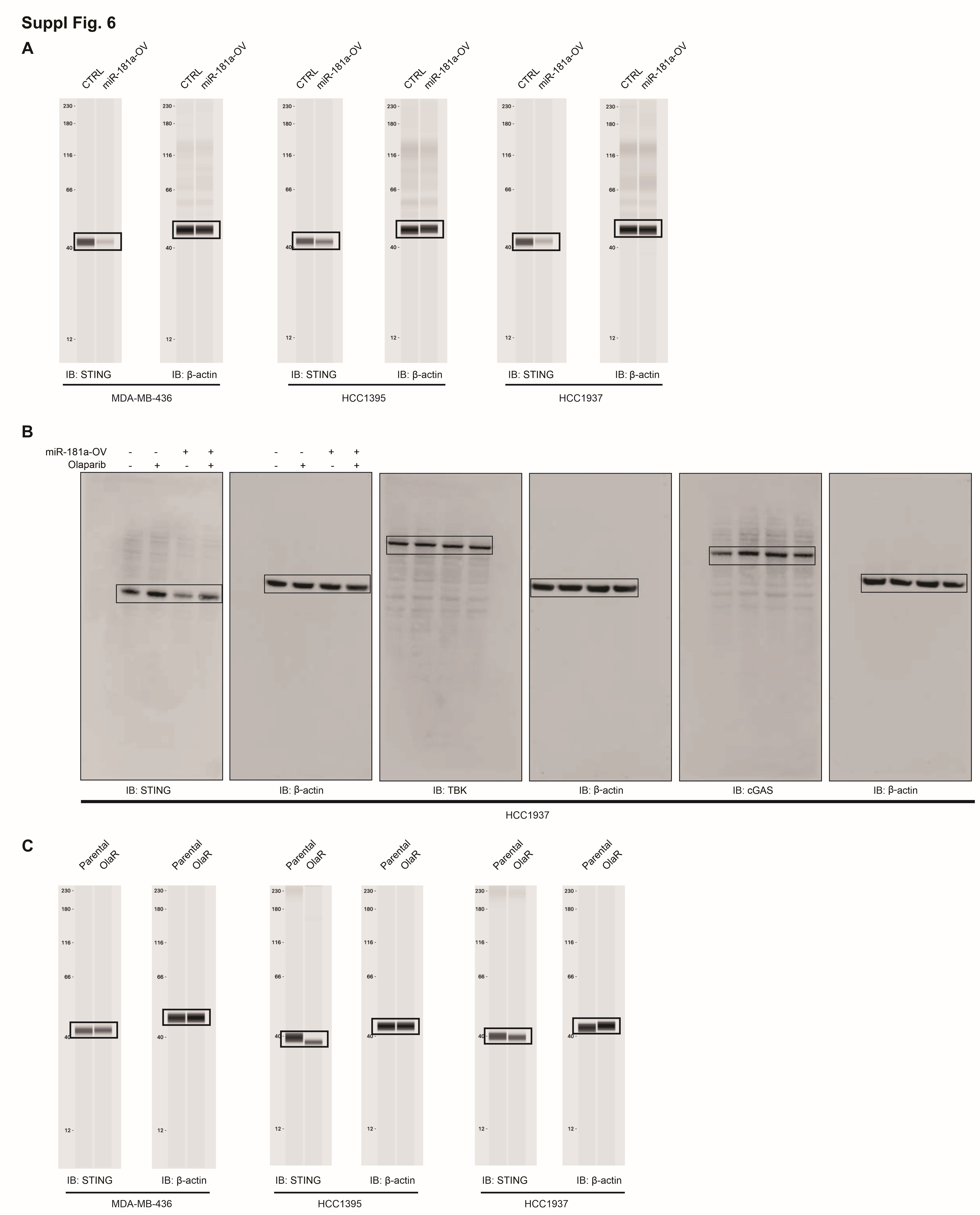


**Figure S6. Uncropped western blotting images. A-C.** Uncropped western blotting images for **Figures 3D** (A), **3H** (B), and **3I** (C) are shown.


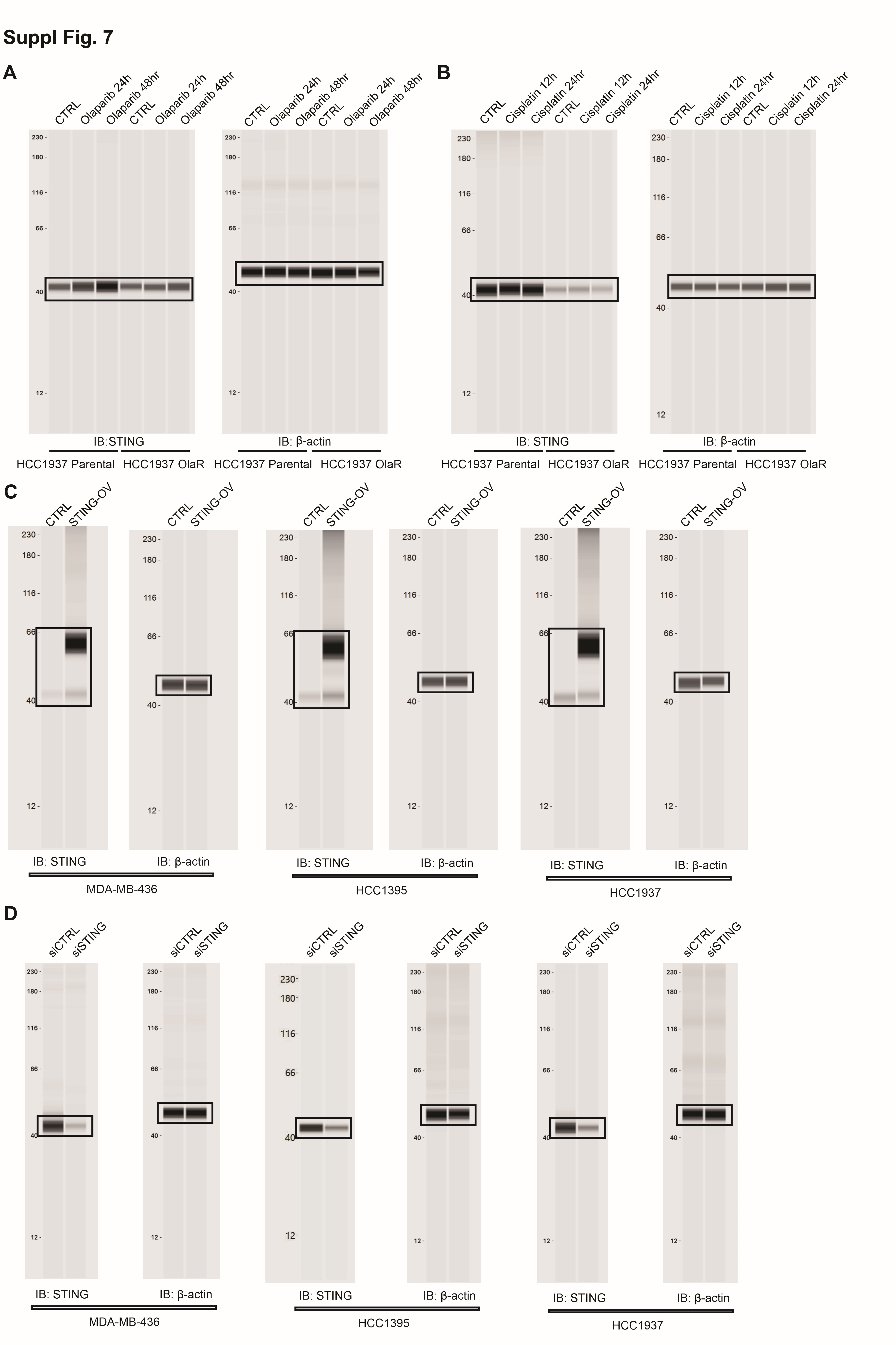


**Figure S7. Uncropped western blotting images. A-D.** Uncropped western blotting images for **Figures 3J** (A), **3K** (B), **4A** (C), and **4E** (D) are shown.


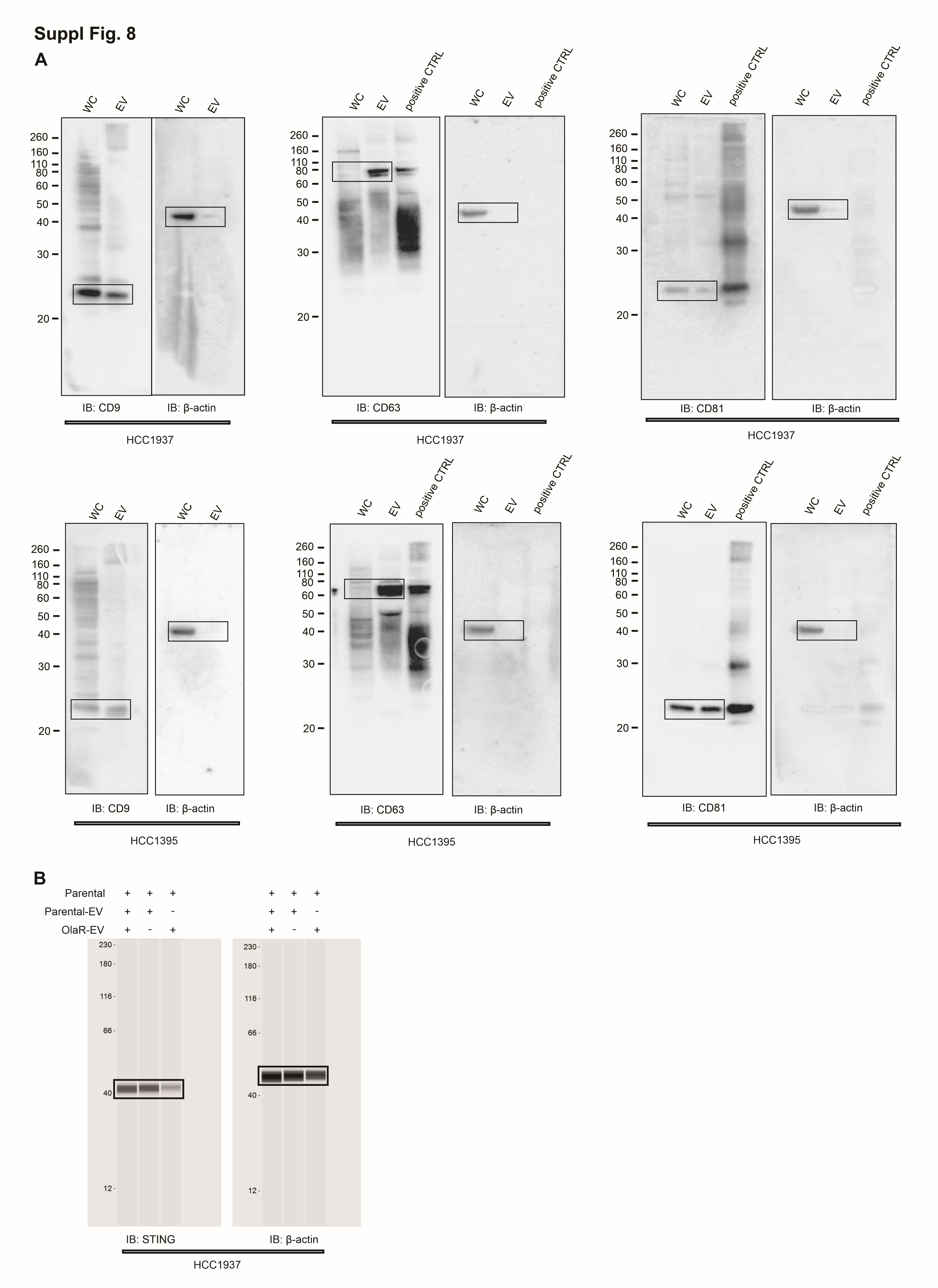


**Figure S8. Uncropped western blotting images. A.** Uncropped western blotting images for **Figure 6A** (A) and **6G** (B)are shown.

**References**

1. Avelar RA, Armstrong AJ, Carvette G, Gupta R, Puleo N, Colina JA*, et al.* Small molecule mediated stabilization of PP2A modulates the Homologous Recombination pathway and potentiates DNA damage-induced cell death. Mol Cancer Ther **2023**

2. Belur Nagaraj A, Joseph P, Ponting E, Fedorov Y, Singh S, Cole A*, et al.* A miRNA-Mediated Approach to Dissect the Complexity of Tumor-Initiating Cell Function and Identify miRNA-Targeting Drugs. Stem Cell Reports **2019**;12:122-34

3. Neviani P, Wise PM, Murtadha M, Liu CW, Wu CH, Jong AY*, et al.* Natural Killer-Derived Exosomal miR-186 Inhibits Neuroblastoma Growth and Immune Escape Mechanisms. Cancer Res **2019**;79:1151-64

4. Wang F, Cerione RA, Antonyak MA. Isolation and characterization of extracellular vesicles produced by cell lines. STAR Protoc **2021**;2:100295

5. Carnell-Morris P, Tannetta D, Siupa A, Hole P, Dragovic R. Analysis of Extracellular Vesicles Using Fluorescence Nanoparticle Tracking Analysis. Methods Mol Biol **2017**;1660:153-73

6. Théry C, Witwer KW, Aikawa E, Alcaraz MJ, Anderson JD, Andriantsitohaina R*, et al.* Minimal information for studies of extracellular vesicles 2018 (MISEV2018): a position statement of the International Society for Extracellular Vesicles and update of the MISEV2014 guidelines. J Extracell Vesicles **2018**;7:1535750

7. Hinestrosa JP, Searson DJ, Lewis JM, Kinana A, Perrera O, Dobrovolskaia I*, et al.* Simultaneous Isolation of Circulating Nucleic Acids and EV-Associated Protein Biomarkers From Unprocessed Plasma Using an AC Electrokinetics-Based Platform. Front Bioeng Biotechnol **2020**;8:581157

8. Ibsen SD, Wright J, Lewis JM, Kim S, Ko SY, Ong J*, et al.* Rapid Isolation and Detection of Exosomes and Associated Biomarkers from Plasma. ACS Nano **2017**;11:6641-51

9. Pusztai L, Yau C, Wolf DM, Han HS, Du L, Wallace AM*, et al.* Durvalumab with olaparib and paclitaxel for high-risk HER2-negative stage II/III breast cancer: Results from the adaptively randomized I-SPY2 trial. Cancer Cell **2021**;39:989-98.e5

10. Ayers M, Lunceford J, Nebozhyn M, Murphy E, Loboda A, Kaufman DR*, et al.* IFN-γ-related mRNA profile predicts clinical response to PD-1 blockade. J Clin Invest **2017**;127:2930-40
